# Precise levels of the *Drosophila* adaptor protein Dreadlocks maintain the size and stability of germline ring canals

**DOI:** 10.1242/jcs.254730

**Published:** 2021-04-27

**Authors:** Kara Stark, Olivia Crowe, Lindsay Lewellyn

**Affiliations:** Department of Biological Sciences, Butler University, Indianapolis, IN 46208, USA

**Keywords:** Dreadlocks, Dock, Egg chamber, Oogenesis, Ring canal

## Abstract

Intercellular bridges are essential for fertility in many organisms. The developing fruit fly egg has become the premier model system to study intercellular bridges. During oogenesis, the oocyte is connected to supporting nurse cells by relatively large intercellular bridges, or ring canals. Once formed, the ring canals undergo a 20-fold increase in diameter to support the movement of materials from the nurse cells to the oocyte. Here, we demonstrate a novel role for the conserved SH2/SH3 adaptor protein Dreadlocks (Dock) in regulating ring canal size and structural stability in the germline. Dock localizes at germline ring canals throughout oogenesis. Loss of Dock leads to a significant reduction in ring canal diameter, and overexpression of Dock causes dramatic defects in ring canal structure and nurse cell multinucleation. The SH2 domain of Dock is required for ring canal localization downstream of Src64 (also known as Src64B), and the function of one or more of the SH3 domains is necessary for the strong overexpression phenotype. Genetic interaction and localization studies suggest that Dock promotes WASp-mediated Arp2/3 activation in order to determine ring canal size and regulate growth.

This article has an associated First Person interview with the first author of the paper.

## Abstract (precis)

**Summary:** *Drosophila* Dock likely functions downstream of WASp and the Arp2/3 complex to regulate the size and stability of the germline ring canals in the developing egg chamber.

## INTRODUCTION

Germ cell development relies critically on the formation of intercellular bridges, which connect developing eggs and sperm to adjacent germ cells or supporting somatic cells in most sexually reproducing organisms. These connections allow for the sharing of organelles, gene products or signaling molecules, which may synchronize or induce specific behaviors, such as division or apoptosis (Greenbaum et al., 2011; Robinson and Cooley, 1996). Defects in the structure or growth of intercellular bridges can lead to infertility, making the study of their structure and regulation essential.

Our knowledge of intercellular bridges is primarily derived from studies done on the germline ring canals of the developing *Drosophila* egg chamber. Egg chamber formation begins with the division of a germline stem cell at the anterior end of the ovariole, called the germarium. One daughter cell, the cystoblast, gives rise to 16 germ cells (one oocyte and 15 nurse cells) by undergoing four rounds of mitotic divisions. At the end of each mitosis, the arrested contractile ring thickens through the accumulation of actin and other proteins; this stable intercellular connection is known as a ring canal (Mahowald, 1971). Once formed, the ring canals undergo a ∼20-fold growth in order to allow the movement of materials from the supporting nurse cells to the oocyte.

About a dozen proteins have been identified that localize to the germline ring canals and regulate their size or stability (Yamashita, 2018). These proteins are organized into an inner and outer rim structure, which must be maintained and anchored to the nurse cell membrane while the ring canals grow. Actin is an abundant component of the inner rim, and during oogenesis, there is a dramatic increase in the number of actin filaments, which is correlated with the significant growth in ring canal diameter (Tilney et al., 1996). The increase in actin filament number and their dynamic behavior likely requires the coordination of multiple actin nucleators and actin-binding proteins, such as HtsRC (produced from the *hts* locus), Kelch and Cheerio (Gerdes et al., 2020; Hudson and Cooley, 2002; Kelso et al., 2002; Robinson et al., 1997; Robinson and Cooley, 1996; Thestrup et al., 2020; Zallen et al., 2002). In addition to actin and actin regulators, a number of kinases are known to localize to ring canals and/or regulate their growth (Dodson et al., 1998; Guarnieri et al., 1998; Hamada-Kawaguchi et al., 2015; Kelso et al., 2002; Kline et al., 2018; Lu et al., 2004; O'Reilly et al., 2006). Two kinases, Btk29 (also known as Btk29A) and Misshapen (Msn), have been shown to impact the localization or modification of adherens junction proteins (Hamada-Kawaguchi et al., 2015; Kline et al., 2018), and a number of mutants that impact adherens junction turnover or the endocytic process lead to ring canal detachment and multinucleation (Bogard et al., 2007; Coutelis and Ephrussi, 2007; Langevin et al., 2005; Loyer et al., 2015; Murthy et al., 2005; Murthy and Schwarz, 2004; Oda et al., 1997; Peifer et al., 1993; Tan et al., 2014; Vaccari et al., 2009). This suggests that changes in the actin cytoskeleton, adherens junctions and membrane trafficking are necessary to maintain ring canal anchoring while also facilitating growth, but how these processes are coordinated during oogenesis is not known.

An attractive candidate to integrate multiple pathways in the germline is the SH2/SH3 adaptor protein Dreadlocks (Dock). Dock and its mammalian homolog Nck (here referring to both NCK1 and NCK2) have been implicated in regulation of the actin cytoskeleton, endocytosis, membrane trafficking and adhesion in many contexts, including immune cell activation and function, cell migration, cancer cell proliferation and invasion, axon guidance and targeting, and cell fusion (Abdallah et al., 2013; Buvall et al., 2013; Chaki and Rivera, 2013; Clemens et al., 1996; Ditlev et al., 2012; Garrity et al., 1996; Joseph et al., 2014; Kaipa et al., 2013; Martin et al., 2020; Ngoenkam et al., 2014; Rivera et al., 2004; Rohatgi et al., 2001). Furthermore, Dock has been shown to localize to germline ring canals during spermatogenesis in the fly (Abdallah et al., 2013). A number of Dock- and Nck-interacting proteins have been identified in other developmental contexts or in different cell types (Chaki et al., 2015; Fan et al., 2003; Hing et al., 1999; Kaipa et al., 2013; Li et al., 2001; Liu et al., 1999; Rivero-Lezcano et al., 1995; Rohatgi et al., 2001; Ruan et al., 1999; Takeuchi et al., 2010; Worby et al., 2001), but a role for Dock in the germline of the developing egg chamber has not been explored. If some of these interactions are conserved, it could place Dock in the unique position to coordinate changes in the actin cytoskeleton with changes in cell–cell adhesion or membrane trafficking to regulate ring canal growth while maintaining stability.

Here, we describe a novel role for Dock in regulation of the germline ring canals in the developing egg chamber. Dock localizes to the ring canals throughout oogenesis. Depletion or mutation of Dock leads to significant changes in ring canal diameter, without significantly impacting ring canal structure or stability; these size changes correlate with a reduction in the levels of HtsRC and actin, which both localize to the inner rim. Overexpression of Dock in the germline leads to catastrophic defects in membrane integrity and ring canal structure. The Dock overexpression phenotype requires ring canal localization, which is mediated through the SH2 domain, as well as the function of at least one of its three SH3 domains. Genetic interaction and localization data suggest that Dock participates in at least two distinct pathways in the germline. Dock likely promotes Arp2/3 activation through recruitment of WASp. In addition, Dock may participate in a separate but related pathway with Msn to regulate ring canal size and/or stability.

## RESULTS

### Dock localizes to germline ring canals throughout oogenesis

**Fig. 1.**
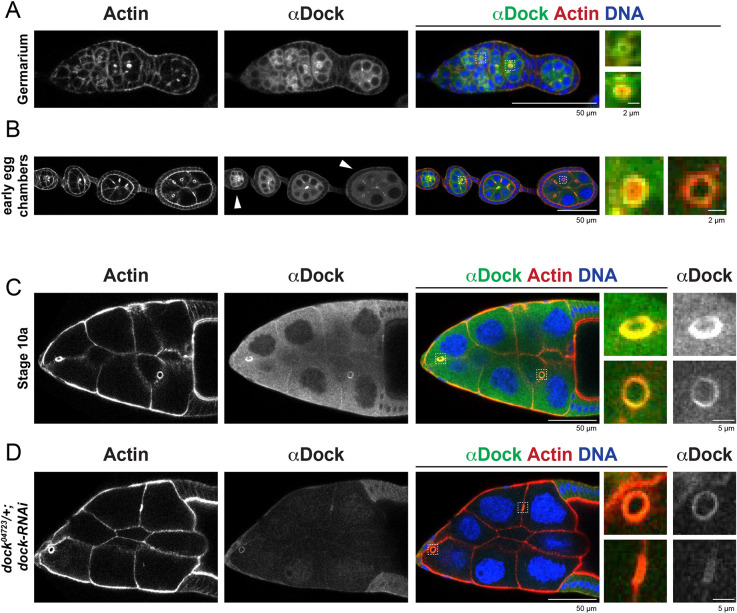
**Dock localizes to the germline ring canals throughout oogenesis.** Single plane confocal images of control (A–C) and *dock^04723^/+; dock-RNAi* (D) egg chambers. Acquisition settings (Dock stain only) for the germarium and younger stages (A,B) were different than those for stage 10 egg chambers (C,D). Arrowheads indicate an example of a young and mid-stage egg chamber that show the progressive reduction in Dock antibody signal.

Because of the established genetic and biochemical interaction of Dock with Msn and other known regulators of the actin cytoskeleton, and its localization to the germline ring canals during spermatogenesis (Abdallah et al., 2013; Kaipa et al., 2013; Ruan et al., 1999), we characterized Dock expression in the female germline. Staining with an anti-Dock antibody (Clemens et al., 1996) revealed the signal is first visible early in the germarium, localizing to newly formed ring canals and in the cytosol (Fig. 1A). The ring canal localization remains throughout oogenesis, although the signal decreases during mid-oogenesis (Fig. 1B). As the cytosolic signal decreases, Dock becomes weakly enriched on nurse cell membranes, which was most obvious in the center of the nurse cell cluster (Fig. 1B). Dock is maintained at ring canals and nurse cell membranes through stage 10 (Fig. 1C), but the levels are dramatically reduced compared to earlier stages, so the acquisition settings had to be altered to see this signal.

To more precisely pinpoint the localization of Dock, we compared it to that of actin, which is found in both the inner rim of the ring canal as well as in microvilli that surround the outer rim (Loyer et al., 2015; Tilney et al., 1996). Early in oogenesis, Dock was peripheral to and partially colocalized with the actin signal (Fig. 1A,B). However, by mid-oogenesis, the Dock and actin signals appeared to largely overlap. Altogether, these data show that Dock levels are regulated over the course of oogenesis and that Dock may function at both the outer and inner rims of the ring canals at different stages.

### Reducing the levels of Dock alters ring canal diameter

**Fig. 2.**
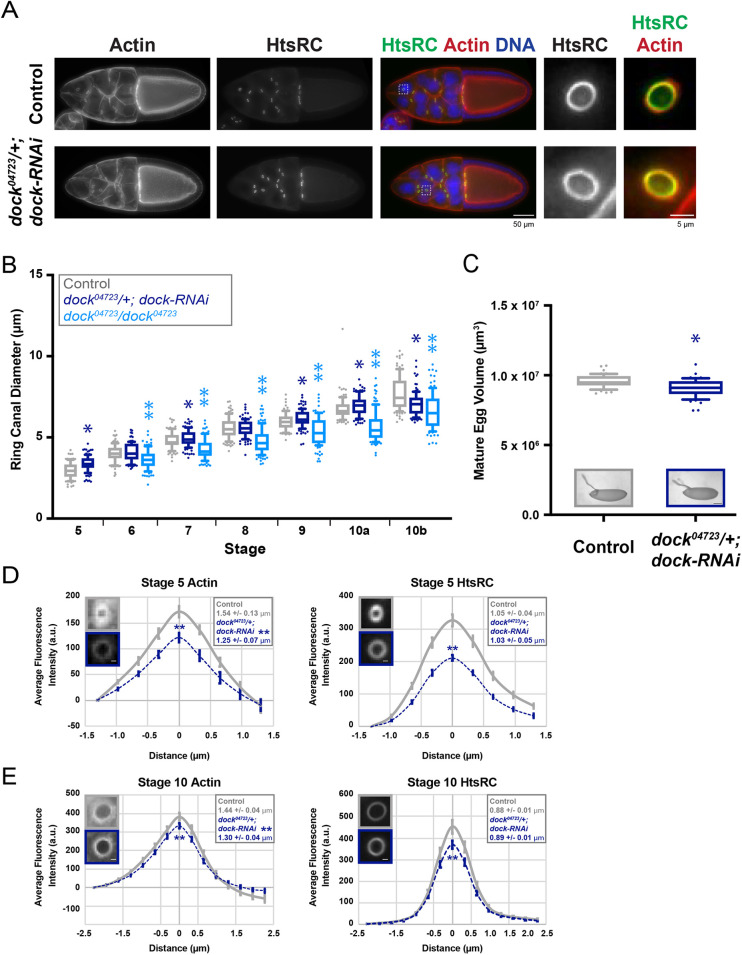
**Dock depletion or mutation alters ring canal diameter and leads to reduced actin and HtsRC.** (A) Maximum intensity projections of fluorescence images of stage 10 control and *dock^04723^/+; dock-RNAi* egg chambers. Boxes indicate ring canals that are shown in the panels to the right. (B) Box and whiskers plot showing the diameter of ring canals connecting nurse cells in control, *dock^04723^/+; dock-RNAi,* and *dock^04723^/dock^04723^* egg chambers. The box represents the 25–75th percentiles, and the median is indicated. The whiskers show the 10–90th percentiles. Individual points represent values outside of that range. *n*=77–164 ring canals per stage and condition. *P<0.05 compared to controls (Kolmogorov–Smirnov test); **P<0.05 compared to *dock^04723^/+; dock-RNAi* and control (one-way ANOVA with Tukey's multiple comparison post-hoc test). (C) Box and whiskers as in B for the volume of mature eggs from control and *dock^04723^/+; dock-RNAi* egg chambers. *n*=55 mature eggs per condition. *P<0.05 compared to control (Kolmogorov–Smirnov test). Representative images of mature eggs for each condition are shown. Scale bar: 100 µm. (D,E) Average fluorescence intensity of phalloidin and HtsRC staining in ring canals of (D) stage 5 and (E) stage 10 control and *dock^04723^/+; dock-RNAi* egg chambers (*n*=33–56). Error bars are s.e.m. Mean±s.e.m. full width at half maximum intensity for each stain at each stage, and representative single plane images are shown. Scale bars: 1 µm (D) and 2 µm (E). **P<0.05 compared to control (unpaired two-tailed *t*-test).

We next tested whether Dock is required for normal ring canal growth and/or stability. We used the maternal triple driver (*otu-GAL4; nanos-GAL4; nanos-GAL4*; hereafter referred to as *MTD-GAL4*) to express a *UAS-dock-RNAi* transgene in the germline throughout oogenesis. To further reduce Dock levels, this depletion was done in combination with a heterozygous mutation (*dock^04723^/+*). Egg chambers were stained with an antibody to HtsRC, which is both necessary and sufficient to recruit F-actin to the inner rim in order to regulate ring canal size and stability (Gerdes et al., 2020; Robinson et al., 1994; Yue and Spradling, 1992). To our surprise, depletion of Dock under these conditions did not cause any obvious defects in ring canal structure or integrity (Figs 1D and 2A); the ring canals always contained a clear lumen and there were no obvious changes to the shape or structure compared to controls. However, there were significant changes in ring canal size during multiple stages of oogenesis; ring canals were significantly larger at stages 5, 7, 9 and 10a, and significantly smaller at stage 10b (Fig. 2B). The mature eggs that developed from the Dock-depleted egg chambers were significantly smaller than controls (Fig. 2C) and showed a reduced rate of hatching (80.5% for *dock^04723^/+; dock-RNAi* vs 93.7% for control). We have seen a similar increase in ring canal diameter using the *UAS-dock-RNAi* transgene alone with multiple different GAL4s (data not shown), suggesting that Dock is necessary to maintain normal ring canal diameter.

To confirm the depletion phenotype, we used the *ovo^D1^* dominant female sterile mutation combined with the FLP/FRT system to generate egg chambers that were homozygous for the *dock* mutation in the germline (*dock^04723^FRT40/dock^04723^ FRT40*) and confirmed that the mutant germ cells contain very little residual Dock protein (Fig. S1A). Interestingly, the average diameter of the ring canals was significantly smaller than both the control and the *dock^04723^/+; dock-RNAi* conditions from stages 6–10b (Fig. 2B). A similar decrease in ring canal diameter was observed for ring canals connecting nurse cells that were homozygous for another mutation, *dock^k13421^* (Fig. S1B). Despite this significant decrease in size, the ring canals always had a normal structure and clear lumen. The stronger phenotype observed in the homozygous mutant germlines led us to question whether there was residual Dock protein in the *dock^04723^/+; dock-RNAi* egg chambers. Staining with the anti-Dock antibody revealed that some Dock-depleted egg chambers still contained Dock protein at the ring canals (Fig. 1D). Therefore, the difference in the RNAi and the mutant phenotype can likely be explained by differences in Dock protein levels, but the underlying cause of this variability in ring canal size is not known. Overall, these data suggest that Dock regulates ring canal size, but is dispensable for their initial formation or structural stability.

To learn more about the size differences that occur when Dock is reduced, we quantified the amount of actin and HtsRC in the ring canals of *dock^04723^/+; dock-RNAi* egg chambers. Depletion of Dock led to a significant reduction in actin and HtsRC at both stage 5 (Fig. 2D) and stage 10 (Fig. 2E). There was also a significant reduction in the width of the actin signal at both stages analyzed (Fig. 2D,E). These data suggest that, although Dock is not essential to recruit actin or HtsRC, it may indirectly regulate actin nucleators or other actin-binding proteins to modulate ring canal size.

### Overexpression of Dock causes defects in ring canal structure and nurse cell multinucleation

**Fig. 3.**
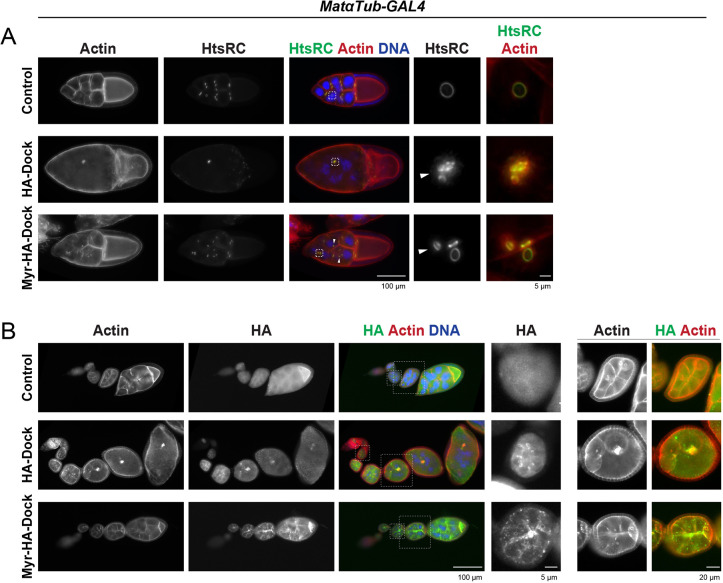
**Dock overexpression causes ring canal collapse and multinucleation.** (A) Maximum intensity projections of fluorescence images of late stage egg chambers. Arrowheads point to abnormally shaped or detached ring canals. (B) Fluorescence images of control, HA–Dock-expressing and Myr–HA–Dock-expressing egg chambers (germarium through to approximately stage 7 or 8). Note that the signal was much brighter for the HA–Dock-expressing egg chambers and that it has to be scaled differently to that for the control and Myr–HA–Dock images. Boxes indicate regions that are shown in panels to the right.

Because Dock levels gradually decrease throughout oogenesis, we next asked whether increasing Dock expression would impact ring canal size or structure. To answer this question, we overexpressed an HA-tagged Dock protein (*UAS-HA-Dock*) in the germline using the *matαTub-Gal4*, which expresses GAL4 beginning at stage 2 of oogenesis. We confirmed a strong overexpression of Dock by western blotting (Fig. S2A). An anti-HtsRC antibody was used to visualize the ring canals (Fig. 3A), and an anti-HA antibody was used to monitor localization of the HA–Dock protein (Fig. 3B). Surprisingly, egg chambers overexpressing Dock contained collapsed or missing ring canals and highly multinucleate egg chambers (Fig. 3A). This phenotype is first obvious during mid-oogensis, but it became progressively worse as the egg chambers developed, leading to the production of very few older egg chambers and a complete absence of mature eggs. The overexpressed Dock protein still localized to the ring canals, at least in early oogenesis (Fig. 3B); it was more challenging to determine the localization in later stages, when the egg chambers were highly abnormal. We found that 67–83% of stage 3–5 egg chambers contained 15 ring canals labeled by HtsRC, which is recruited after the end of the mitotic divisions. Furthermore, because this GAL4 is not expressed until after the mitotic divisions are complete, this result suggests that the strong phenotype is not due to defects in incomplete cytokinesis, but that increasing the amount of Dock protein after ring canal formation has catastrophic consequences in the germline.

Dock primarily localizes to the ring canals, but we did observe weak staining at the nurse cell membranes, which was most obvious in the center of the nurse cell cluster. The Msn kinase similarly localizes to both the ring canals and nurse cell membranes, and tethering Msn to the membrane causes ring canal detachment and multinucleation (Kline et al., 2018). In egg chambers expressing the membrane-tethered Myr–MsnWT, Dock is ectopically enriched on nurse cell membranes (Fig. S2C). The kinase activity of Msn was not required for this localization, as expression of the kinase-inactive membrane-tethered Msn (*UAS-Myr-MsnKR*; Kline et al., 2018) caused an even higher enrichment of Dock on nurse cell membranes (Fig. S2C). These data suggest that Msn is able to recruit Dock in the germline.

To ask whether Dock can similarly recruit Msn, we used *matαTub-GAL4* to overexpress an HA-tagged form of Dock containing an N-terminal myristoylation tag that tethers it to the membrane (*UAS-Myr-HA-Dock*). Staining with an anti-HA antibody revealed that the Myr–HA–Dock localized strongly to nurse cell membranes and ring canals as well as to large puncta in the cytosol (Fig. 3B). These egg chambers showed an enrichment of an endogenously tagged Msn–YFP at nurse cell membranes and abnormal actin-containing structures (Fig. S2D). Therefore, Msn and Dock are capable of recruiting each other to ring canals and nurse cell membranes when overexpressed.

Expression of Myr–Dock also caused ring canal collapse and the formation of multinucleate nurse cells (Fig. 3A). This phenotype was similar to that observed in Myr–MsnWT-expressing egg chambers, and it suggests that, when tethered to the membrane, Msn and Dock can function together to destabilize ring canals and nurse cell membranes. However, the Myr–Dock phenotype was less severe than that seen upon expression of the non-membrane tethered HA–Dock (Fig. 3A,B). Because these transgenes were not integrated into the same chromosomal location, we cannot exclude the possibility that the variation in the phenotype is due to differences in transgene expression. Alternatively, tethering Dock to the membrane could reduce phenotype severity by partially sequestering Dock-interacting proteins away from the ring canals.

### The SH2 domain localizes Dock to ring canals, whereas the SH3 domain(s) mediate the overexpression phenotype

**Fig. 4.**
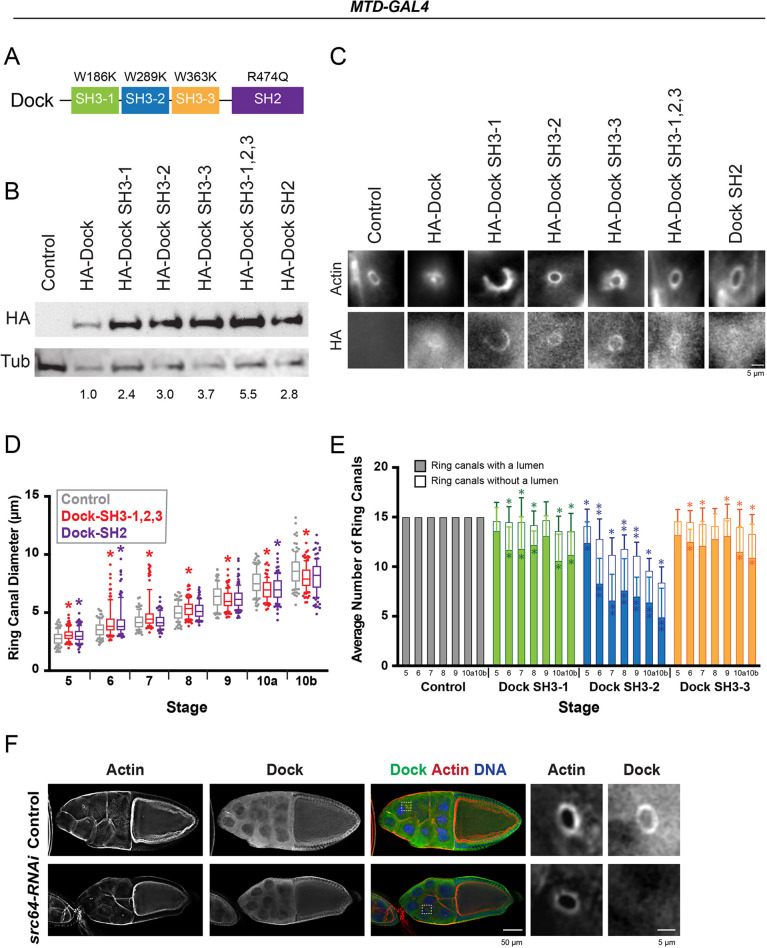
**Ring canal localization and SH3 domain function are required for the overexpression phenotype.** (A) Schematic of Dock domain structure and point mutations. (B) Western blot of whole-ovary lysate. Relative expression levels of HA–Dock are indicated. (C) Average intensity projections of fluorescence images of ring canals from control egg chambers and egg chambers overexpressing HA–Dock transgenes. Autoscaling was used to best visualize the signal in each condition. (D) Box and whiskers plot for the diameter of ring canals connecting nurse cells. The box represents the 25–75th percentiles, and the median is indicated. The whiskers show the 10–90th percentiles. Individual points represent values outside of that range. *n*=88–121 ring canals per stage and condition. *P<0.05 compared to controls (Kolmogorov–Smirnov test). (E) Average number of visible ring canals with and without lumens in the indicated conditions. Error bars represent s.d. *n*=10 egg chambers per stage and condition. *P<0.05 for total number of ring canals with or without a lumen compared to controls; **P<0.05 from control, Dock SH3-1 and Dock SH3-3 (one-way ANOVA with Tukey's multiple comparison post-hoc). Owing to the severity of the phenotype, we were not able to accurately count the number of ring canals in the HA–Dock-overexpressing egg chambers. (F) Stage 10 control and *src64-RNAi* egg chambers. Boxes indicate regions that are shown in panels to the right.

To further dissect the Dock overexpression phenotype, key residues in each of four binding domains of Dock were mutated. Transgenes containing mutations in the SH2, SH3-1, SH3-2, SH3-3 or a ‘triple mutant’ containing substitutions in all three SH3 domains (SH3-1,2,3) (Fig. 4A) were generated and integrated into the same chromosomal location using the PhiC31 integrase. They were then overexpressed in the female germline using *MTD-GAL4*. Western blot analysis of whole-ovary lysate (Fig. 4B; Fig. S3A) or immunofluorescence (Fig. 4C) using an anti-HA antibody confirmed expression of all transgenes. Expression of HA–Dock SH2 or HA–Dock SH3-1,2,3 did not have a significant effect on ring canal structure or integrity (Fig. 4C; Fig. S3B). The egg chambers always contained the expected number of ring canals (11 connecting nurse cells and four connecting the nurse cells to the oocyte), and those ring canals always contained a clear lumen that would presumably support cytoplasmic transfer to the oocyte. However, we did observe a significant difference in average ring canal diameter at many stages for both conditions (Fig. 4D), and the mature egg volumes were significantly reduced (Fig. S4A). There was only a modest reduction in embryo viability (84.6% for HA–Dock SH2, 93.9% for HA–Dock SH3-1,2,3, and 95.1% for control). Expression of HA–Dock SH3-1, HA–Dock SH3-2 or HA–Dock SH3-3 caused a moderate amount of abnormal ring canals and nurse cell multinucleation (Fig. 4C,E; Fig. S3B), which distinguished these conditions from the more severely affected HA–Dock-expressing egg chambers. The milder phenotype allowed us to observe a range of abnormal ring canal structures. For example, the ring canals shown in the HA–Dock SH3-1- and HA–Dock SH3-3-expressing egg chambers were only partial rings, which were highly enriched in actin (Fig. 4C), which suggests that overexpression of Dock may recruit one or more interactors that promote actin polymerization or inhibit turnover. Because of the frequency of abnormal ring canals, we did not attempt to measure their diameter. We found that 64–90% of stage 4–5 egg chambers expressing HA–Dock, HA–Dock SH3-1, HA–Dock SH3-2 or HA–Dock SH3-3 contained 15 ring canals marked by HtsRC. However, because we used *MTD-GAL4*, which expresses GAL4 throughout oogenesis, we cannot rule out that an earlier defect in cytokinesis could be contributing to these phenotypes.

Of the three single SH3 domain point mutants, the overexpression of HA–Dock SH3-2 produced the strongest phenotype, characterized by fewer ring canals overall and a higher number of lumen-less ring canals (Fig. 4E). The mature eggs produced from the HA–Dock SH3-2-expressing egg chambers were smaller, although not significantly, than those from HA–Dock SH3-1 and HA–Dock SH3-3 (Fig. S4A). However, the embryonic viability was similar for the mature eggs produced from the SH3 domain single mutants (31.4% for HA–Dock SH3-1, 32.7% for HA–Dock SH3-2, 27.1% for HA–Dock SH3-3, and 95.1% for control), so the relative importance of the individual SH3 domains remains unclear.

Immunofluorescence revealed different domain-specific requirements for Dock localization. Mutation of the SH2 domain eliminated the HA–Dock signal at the ring canals and led to a relatively higher cytosolic signal. By contrast, mutation of the SH3 domains did not completely eliminate localization (Fig. 4C), although the HA–Dock SH3-1,2,3 was less enriched than some of the other Dock proteins. From these data, we conclude that the SH2 domain is required for Dock to localize to the germline ring canals.

SH2 domains bind to phosphotyrosine (pTyr) (Lim and Pawson, 2010; Pawson, 1995; Songyang et al., 1993), and this domain localizes Dock to ring canals during spermatogenesis in *Drosophila* (Abdallah et al., 2013). In the egg chamber, a pTyr residue localizes to the outer rim of the ring canals beginning in region 1 of the germarium, and its presence depends on the activity of the Src64 and/or Btk29 kinases (Dodson et al., 1998; Guarnieri et al., 1998; Hamada-Kawaguchi et al., 2015; Lu et al., 2004; Roulier et al., 1998). Interestingly, depletion of Src64 reduced the enrichment of Dock at the ring canals (Fig. 4F; Fig. S4B), suggesting that the Src64-generated pTyr epitope recruits Dock to this location.

### Reducing the levels of WASp or the Arp2/3 complex subunit Arp3 partially rescued the Myr–Dock overexpression phenotype

Because many Dock- and Nck-interacting proteins have been identified, we took a candidate-based approach to determine the molecular basis of the Dock overexpression phenotype. We hypothesized that Dock overexpression recruits an excess of one or more of its interactors. If so, then reducing the levels of the interactor should at least partially rescue the phenotype. Owing to the strength of HA–Dock overexpression, we looked for suppression of the milder Myr–HA–Dock phenotype.

As Msn and Dock can recruit each other to nurse cell membranes (Fig. S2C,D), we first tested whether reducing Msn levels could rescue the Myr–Dock expression phenotype. We found that there was no significant change in the total number of ring canals or in the number of ‘abnormal’ (collapsed, lumen-less or detached) ring canals during stages 9 to 10b compared to what is seen with Myr–Dock expression alone (Fig. S5). Although it is possible that a stronger reduction in Msn levels is necessary to observe a phenotypic rescue, these data suggest that the Myr–Dock overexpression phenotype is not largely due to ectopic recruitment of Msn.

**Fig. 5.**
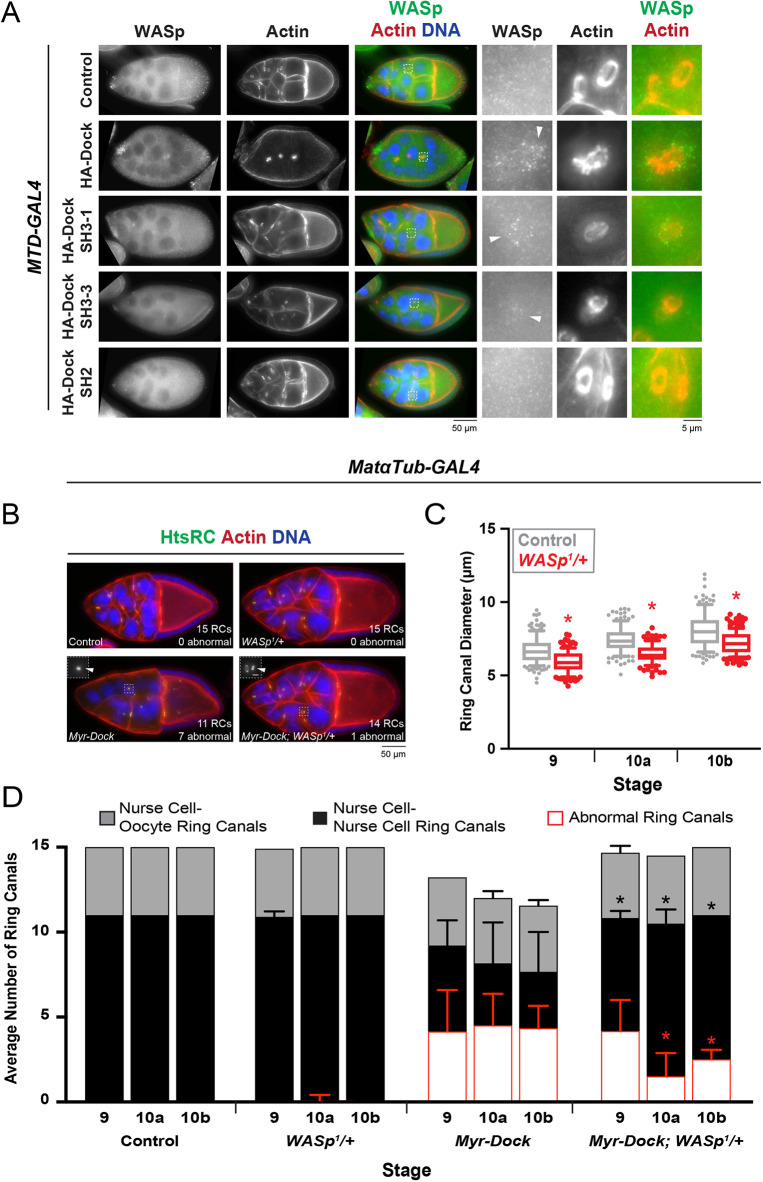
**Reducing the levels of WASp partially rescues the Myr-Dock expression phenotype.** (A) Maximum intensity projections of fluorescence images of stage 9 control and HA–Dock-expressing egg chambers. Arrowheads point to modest enrichment of WASp near actin associated with abnormally shaped ring canals. (B) Maximum intensity projections of fluorescence images of stage 10a egg chambers. Box indicates region that is shown in the inset. Scale bar in inset is 5 µm. Arrowheads indicate examples of abnormal ring canals. (C) Box and whiskers plot for the diameter of ring canals connecting nurse cells in control and *WASp^1^/+* egg chambers. The box represents the 25–75th percentiles, and the median is indicated. The whiskers show the 10–90th percentiles. Individual points represent values outside of that range. *n*=108–165 ring canals per stage and condition. *P<0.05 (Kolmogorov–Smirnov test). (D) Average number of visible ring canals for the conditions shown in B. Graph also shows the average number of ‘abnormal’ ring canals, most of which were originally connecting nurse cells. Error bars represent s.d. *n*=4–14 egg chambers per stage and condition. *P<0.05 for the number of ring canals connecting nurse cells (black) or the number of collapsed ring canals (red) compared to *Myr-Dock* alone (unpaired two-tailed *t*-test).

Another protein that interacts with Dock is the Arp2/3 activator WASp (Kaipa et al., 2013; Worby et al., 2001). Although staining for WASp in the germline is very weak, expression of multiple HA–Dock transgenes led to the modest accumulation of WASp near the actin that was enriched around some of the abnormally shaped ring canals (Fig. 5A). Therefore, we tested whether reducing WASp levels could rescue the Myr–Dock phenotype. Egg chambers that were heterozygous for the *WASp^1^/+* mutation typically contained the expected number of ring canals (Fig. 5B,D); however, they were significantly smaller than controls in the stages analyzed (Fig. 5C). When WASp levels were then reduced in the Myr–Dock-expressing egg chambers with the same heterozygous mutation, there was a significant decrease in the number of ‘abnormal’ ring canals at stages 10a and 10b, and a significant increase in the total number of ring canals connecting nurse cells at all stages analyzed; we could typically locate all 15 (Fig. 5B,D). Introducing heterozygous mutations in both *msn* and *WASp* in the Myr–Dock-expressing egg chambers did not produce a stronger phenotypic rescue (Fig. S5). This suggests that the ectopic recruitment of WASp significantly contributes to the Myr–Dock phenotype.

**Fig. 6.**
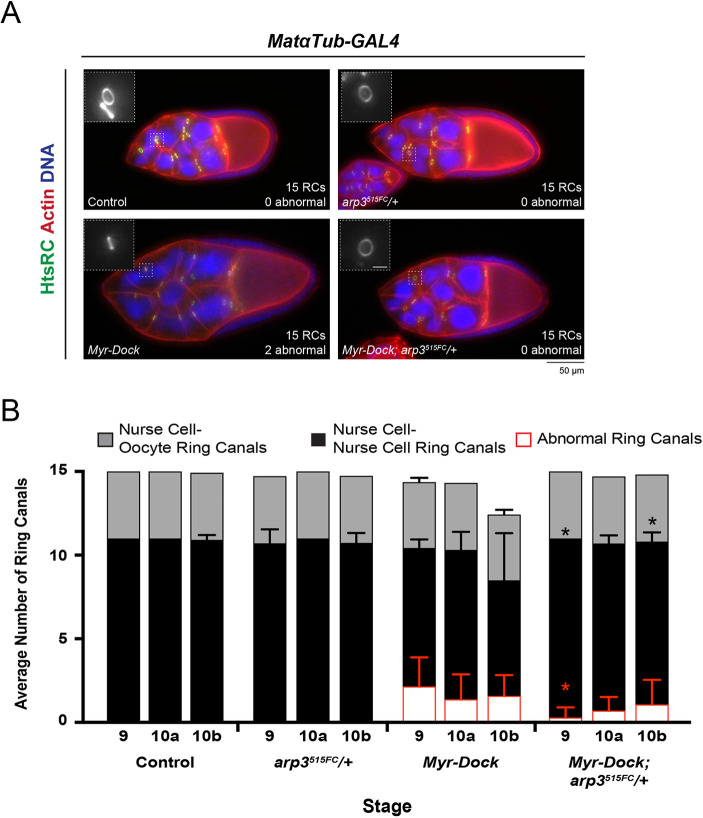
**Reducing the levels of the Arp2/3 complex subunit, Arp3, also partially rescues the Myr-Dock expression phenotype.** (A) Maximum intensity projections of fluorescence images of late stage 9 egg chambers. Scale bar for the inset is 5 µm. (B) Average number of visible ring canals connecting nurse cells and nurse cells to the oocyte. Graph also shows the average number of ‘abnormal’ ring canals, most of which were originally connecting nurse cells. Error bars represent s.d. *n*=10–16 egg chambers per stage and condition. *P<0.05 in the number of ring canals connecting nurse cells (black) or the number of collapsed ring canals (red) compared to *Myr-Dock* alone (unpaired two-tailed *t*-test).

Because WASp activates the Arp2/3 complex, we next tested whether reducing the levels of an essential subunit of the complex would provide a similar rescue. We introduced a heterozygous mutation in Arp3 to the Myr–Dock-expressing egg chambers and found that there was a significant decrease in the number of ‘abnormal’ ring canals at stage 9, and the *arp3^515FC^/+; Myr-Dock* egg chambers contained significantly more ring canals connecting nurse cells compared to *Myr-Dock* egg chambers at stage 9 and 10b (Fig. 6A,B). Although again this rescue was not complete, it provides further evidence that Dock can recruit WASp and positively regulate the Arp2/3 complex, and that ectopic Arp2/3 activation could lead to ring canal collapse and nurse cell multinucleation.

### Reducing Dock levels enhances the *arpC2-RNAi* phenotype, but not the *msn-RNAi* phenotype

As reducing WASp or Arp3 levels led to a significant, although partial, rescue of the Myr–Dock overexpression phenotype, it suggests that Dock could recruit WASp to the ring canals to promote Arp2/3 complex activation under normal conditions. We recently demonstrated that strong depletion of the essential Arp2/3 complex subunit ArpC2 in the germline using *MTD-GAL4* leads to the formation of small, and sometimes lumen-less, ring canals (Thestrup et al., 2020); a similar decrease in ring canal diameter was observed in the *dock/dock* mutant germ cells (Fig. 2B; Fig. S1B). Furthermore, *dock^04723^/+; dock-RNAi* egg chambers showed reduced actin specifically at ring canals (Fig. 2D,E), which suggests that Dock could positively regulate actin nucleation.

**Fig. 7.**
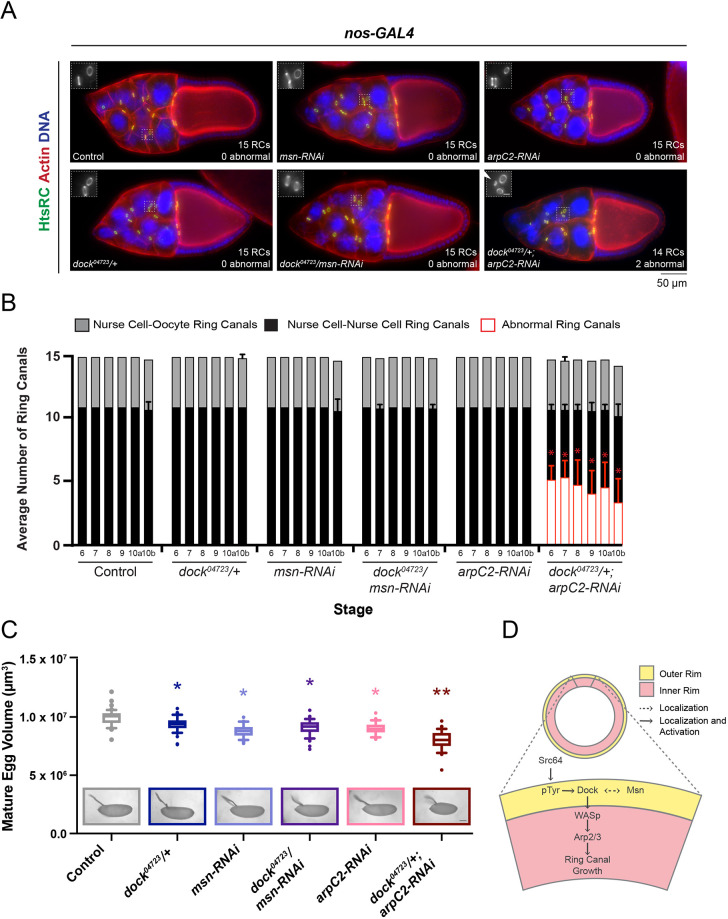
**Reducing Dock enhances the *arpC2-RNAi* phenotype.** (A) Maximum intensity projections of fluorescence images of stage 10 egg chambers. Box indicates region shown in the inset. Scale bar for the inset is 5 µm. Arrowhead indicates a collapsed ring canal. (B) Average number of visible ring canals in each condition. Graph also shows the average number of ‘abnormal’ ring canals; all collapsed ring canals originated from ring canals connecting nurse cells. Error bars represent s.d. *n*=10 egg chambers per stage and condition. *P<0.01 compared to *arpC2-RNAi* alone (unpaired two-tailed *t*-test). (C) Box and whiskers plot for the volume of mature eggs in each condition. The box represents the 25–75th percentiles, and the median is indicated. The whiskers show the 10–90th percentiles. Individual points represent values outside of that range. *n*=43–53 mature eggs per condition. *P<0.05 compared to control; **P<0.05 compared to both control and the RNAi alone (one-way ANOVA with Tukey's multiple comparison post-hoc test). Representative images of mature eggs are shown. Scale bar: 100 µm. (D) Summary of the proposed interactions between the proteins studied.

To test this model, we further explored the genetic interaction between Dock and the Arp2/3 complex. Performing a weaker depletion of ArpC2 using *nos-GAL4*, which expresses GAL4 in two pulses during oogenesis, did not dramatically alter ring canal structure (Fig. 7A). From stages 6–10b, all *arpC2-RNAi* egg chambers analyzed contained the expected number of ring canals, none of which were collapsed or lumen-less (Fig. 7A,B). However, the mature eggs that developed from these egg chambers were significantly smaller than control (Fig. 7C). In contrast, when Dock levels were reduced in the *arpC2-RNAi* background using a heterozygous mutation (*dock^04723^/+*), the egg chambers contained an average of ∼3­–5 ‘abnormal’ ring canals from stages 6–10b (Fig. 7A,B), and the mature eggs that developed were significantly smaller than the control or either single manipulation (*dock^04723^/+* or *arpC2-RNAi* alone; Fig. 7C). By contrast, reducing Dock levels did not enhance a weak *msn-RNAi* phenotype (Fig. 7A–C). These findings suggest that Dock recruits WASp to ring canals to positively regulate the Arp2/3 complex to determine ring canal size and maintain stability (Fig. 7D). Additionally, although they may influence the localization of each other (Fig. 7D), Dock and Msn likely do not promote the activity of the other.

## DISCUSSION

We have demonstrated a novel role for the adaptor protein Dreadlocks (Dock) in the germline of the developing *Drosophila* egg chamber. Dock localizes to germline ring canals throughout oogenesis (Fig. 1), and its levels and localization must be precisely controlled to allow oogenesis to progress normally. Germline depletion or mutation of Dock caused a significant change in ring canal diameter without affecting overall structure or stability (Fig. 2; Fig. S1), whereas overexpression or expression of a membrane-tethered form of Dock led to ring canal collapse or detachment and nurse cell multinucleation (Fig. 3). Dock localizes to the ring canals through its SH2 domain, and one or more of its SH3 domains likely recruit interacting proteins to this location (Fig. 4). Although Msn can be ectopically enriched by Myr–Dock in the germline, this is not the primary cause of the overexpression phenotype (Figs S2D and S5). Instead, genetic interaction data suggest that Dock positively regulates actin levels at the ring canal through the WASp-mediated activation of the Arp2/3 complex (Figs 5,6 and 7), but it may participate in additional pathways to regulate ring canal size and stability in the germline.

### Specific domains are required for Dock localization and function at the ring canal

Dock is one of only a few proteins known to localize to the outer rim of the ring canal along with the glycoprotein, mucin-D, and an unidentified tyrosine-phosphorylated protein, whose modification depends on the activity of two kinases, Btk29 and Src64 (Dodson et al., 1998; Guarnieri et al., 1998; Lu et al., 2004; Roulier et al., 1998; Kramerova and Kramerov, 1999). The SH2 domain is required to localize the HA–Dock protein to the ring canals, and depletion of the Src64 kinase reduced Dock localization (Fig. 4). Because SH2 domains bind to pTyr residues (Lim and Pawson, 2010; Pawson, 1995; Songyang et al., 1993), this provides a clear mechanism by which Dock could be recruited to the outer rim early in oogenesis, downstream of the activity of Btk29 and/or Src64. Furthermore, because the SH2 domain and pTyr also mediate the localization of Dock during spermatogenesis (Abdallah et al., 2013), this mechanism of Dock localization may be conserved during gametogenesis. The outer rim may serve as a bridge between the inner rim, which is likely more dynamic in composition, and the plasma membrane and actin-rich microvilli, which are thought to anchor the ring canals to the membrane (Loyer et al., 2015; Tilney et al., 1996). Therefore, from this location, Dock could play an important role in coordinating changes in the actin-based structures (the inner rim and microvilli) with changes in membrane composition or adherens junctions.

Ring canal localization and the function of one or more of the SH3 domains is essential for the strong overexpression phenotype. Overexpression of HA–Dock SH3-1,2,3, which contains point mutations in all three SH3 domains, had only a modest effect on ring canal size, mature egg size and embryonic viability (Fig. 4, Figs S3B and S4A). Interestingly, overexpression of HA–Dock SH3-2 caused a more severe ring canal phenotype than expression of either HA–Dock SH3-1 or HA–Dock SH3-3. This suggests that the function of the SH3-1 and SH3-3 domains may be more important in recruiting effectors that cause the dramatic nurse cell defects. However, the effect of expression of HA–Dock SH3-2 is still not as severe as that of expression of HA–Dock, suggesting that the SH3-2 does contribute to the phenotype.

It is possible that multiple SH3 domains are required for Dock to interact with certain proteins. For example, the function of all three SH3 domains has been shown to be required for strong Dock–WASp interaction in a yeast two-hybrid and immunoprecipitation experiments (Kaipa et al., 2013; Worby et al., 2001). Although we still see some enrichment of WASp in egg chambers overexpressing single SH3 domain mutants (Fig. 5A), mutating any one of the three domains may reduce recruitment of WASp to the ring canals, thereby reducing the severity of the phenotype. Yeast two-hybrid assays suggest that the SH3-1 and SH3-2 domains, but not the SH3-3 domain, are important to mediate the Dock–Msn interaction (Ruan et al., 1999). If specific domains are capable of interacting with multiple different proteins, it suggests that there could be competition between different interactors for binding to Dock at the ring canals, and some of the depletion and overexpression phenotypes that we observe could be due to imbalances in these interactions. Further experiments will be required to identify additional domain-specific Dock-interacting proteins, and to assess their contribution to loss-of-function or overexpression phenotypes.

Our data suggest that Dock binding to pTyr is essential for ring canal localization, but there are likely other regulatory mechanisms that determine Dock levels in the germline. Immunofluorescence demonstrated a very high level of Dock protein in the cytosol in the germarium and during early oogenesis, but this signal is reduced as development progresses (Fig. 1). This suggests that there could be two populations of Dock – a cytosolic pool that is subject to developmentally controlled degradation, and a potentially more stable population at the ring canal. Nck is ubiquitylated by the E3 ligase, Cbl-b. This ubiquitylation and subsequent degradation is likely mediated by the SH3 domains, as mutation of any of the three reduced the interaction with Cbl-b and led to the ectopic accumulation of Nck (Joseph et al., 2014). Interestingly, Kelch, which localizes to germline ring canals, functions as part of a Cullin3 RING ubiquitin ligase complex, CRL3, to target the degradation of HtsRC at the ring canal (Hudson et al., 2019, 2015; Hudson and Cooley, 2010; Kelso et al., 2002; Robinson et al., 1994; Xue and Cooley, 1993). Although Dock was not identified biochemically as a substrate for this Kelch-containing CRL3 complex (Hudson et al., 2019), another substrate adaptor or ligase could target Dock turnover in the germline; if overexpression of HA–Dock overwhelms this system, it could explain the severity of the phenotype.

It will also be interesting to determine the role for the N-terminus of the Dock protein in its localization and function. Expression of a shorter isoform of Dock (isoform A), which lacks the first 138 amino acids, caused modest defects in ring canal structure (Fig. S2B). Interestingly, although this shorter isoform appears to be expressed in ovary lysate (Fig. S2A), and would be expected to contain the SH2 domain, it did not show strong localization to the ring canals (Fig. S2B). This suggests that the longer isoform is likely the one functioning at the ring canal, but additional experiments will be necessary to determine whether isoform A is normally expressed in the germline, and if so, whether it may regulate one or more interacting proteins in the cytosol during oogenesis.

It will also be of interest to further explore the progression of the Dock overexpression phenotype. When HA–Dock is expressed after the completion of germ cell divisions using *Matαtub-GAL4* (Fig. 3), the observed phenotypes likely arise through destabilization of ring canals after they have formed, but the mechanism underlying this phenotype remains unclear*.* However, when HA–Dock is expressed using *MTD-GAL4*, there are a number of egg chambers that contained more than the expected 15 nurse cells; this phenotype was not observed in egg chambers expressing any of the other HA–Dock transgenes using *MTD-GAL4*. Further studies will be required to determine whether this is due to a packaging defect or misregulation of germ cell divisions within the germarium.

### Dock likely acts upstream of the Arp2/3 complex to promote ring canal growth

Dock likely promotes ring canal growth indirectly through activation of the Arp2/3 complex. Depletion of the Arp2/3 subunit ArpC2 leads to the formation of small lumen-less ring canals (Thestrup et al., 2020). Although depletion or mutation of Dock on its own never resulted in lumen-less ring canals, we found a significant reduction in both actin and HtsRC in these conditions (Fig. 2), suggesting that Dock may regulate the activity of an actin nucleator or other type of actin regulator. Furthermore, reducing Dock levels significantly enhanced a weak *arpC2-RNAi* phenotype (Fig. 7), which suggests that the two could function in the same pathway. Although the larger ring canals observed at earlier stages in the *dock^04723^/+; dock-RNAi* egg chambers (Fig. 2) were initially puzzling, it was reminiscent of aspects of the *arpC2-RNAi* phenotype. In a previous study, when ArpC2 was depleted in the germline, we saw an increase in ring canal diameter in the germarium; at later stages, for ring canals that did contain a clear lumen, the average diameter was often larger than controls (Thestrup et al., 2020). This suggests that the Arp2/3 complex could play an early role in promoting contractile ring closure during incomplete cytokinesis and/or limit ring canal growth during later stages. If Dock functions upstream of the Arp2/3 complex at multiple stages of oogenesis, this could explain the variation in ring canal size that we observe. Alternatively, reducing Dock levels could lead to modest reductions in Arp2/3 activity, which slightly destabilizes the ring canal, leading to expansion, whereas stronger reductions could inhibit growth. Depletion of the Dock homolog Nck leads to reduced myosin activity (Chaki et al., 2013), so it is also possible that the increase in ring canal size is due to reduced myosin-based contractility. Additional work will be required to distinguish between these possibilities.

Our data suggest that Dock promotes Arp2/3 activation indirectly through recruitment of WASp. In egg chambers overexpressing various HA–Dock transgenes, we observed ectopic recruitment of WASp near abnormally shaped ring canals (Fig. 5A). This was surprising given that WASp does not normally localize to germline ring canals (Rodriguez-Mesa et al., 2012), and germline mutation of *WASp* did not produce any obvious structural defects (Zallen et al., 2002). However, we observed a significant decrease in ring canal diameter in *WASp^1^/+* heterozygous mutant egg chambers compared to control (Fig. 5C), similar to the *dock* mutant phenotype (Fig. 2; Fig. S1B). This suggests that if Dock does normally recruit WASp to germline ring canals, the levels could be so low or transient that they are difficult to detect through immunofluorescence. The local WASp-mediated Arp2/3 activation may contribute to the control of ring canal size, but it is not essential for structural integrity. However, if WASp is ectopically recruited to ring canals and/or nurse cell membranes through overexpression of Dock, this can lead to catastrophic defects in the germline. This connection is consistent with what has been seen in other systems. Antibody-induced clustering of the three SH3 domains of Nck in NIH-3T3 cells led to the ectopic accumulation of WASp and the formation of actin tails (Rivera et al., 2004), which was very similar to what we observed in egg chambers overexpressing HA–Dock (Fig. 4). Therefore, interaction with Dock may bring WASp to the ring canal to increase the local activity of the Arp2/3 complex.

WASp-mediated activation of Arp2/3 is typically associated with endocytosis. Although the specific role for endocytosis in regulation of ring canals is unknown, many mutations that affect membrane trafficking or the endocytic process produce a similar set of defects as those seen in egg chambers overexpressing Dock – ring canal detachment or collapse and nurse cell multinucleation (Bogard et al., 2007; Coutelis and Ephrussi, 2007; Langevin et al., 2005; Loyer et al., 2015; Murthy et al., 2005; Murthy and Schwarz, 2004; Oda et al., 1997; Peifer et al., 1993; Tan et al., 2014; Vaccari et al., 2009). In addition, when Dock was tethered to the membrane, it caused Msn–YFP and actin to accumulate into large cytosolic puncta (Fig. S2D), which could indicate that endocytosis has been disrupted. Therefore, it is possible that Dock overexpression and ectopic WASp recruitment disrupt the normal balance between endocytosis and secretion that is required to maintain ring canal anchoring and membrane integrity, especially during periods of dramatic tissue growth.

One potential target of the endocytic pathway could be to regulate the turnover of adherens junctions. The presence of adherens junctions in the germline is essential to anchor ring canals to the membrane and prevent nurse cell multinucleation (Loyer et al., 2015; Oda et al., 1997; Peifer et al., 1993). Although adherens junction dynamics have not been directly measured in the germline, there is evidence suggesting that their turnover facilitates ring canal growth (Hamada-Kawaguchi et al., 2015). We have recently shown that altering the levels of Msn leads to changes in ring canal size, which correlate with changes in E-cadherin localization, and in some cases, reduced phosphorylation of β-catenin (Kline et al., 2018). When tethered to the membrane, Msn and Dock can ectopically enrich each other (Fig. S2C,D), which suggests that the two could function together in some capacity. Additional studies will be required to explore whether Dock and Msn regulate adherens junction stability or turnover in the germline, and also to determine the nature of this interaction.

It is also possible that Dock recruits other interactors to the ring canal to activate the Arp2/3 complex or otherwise impact actin structure or dynamics. For example, Dock can physically interact with another Arp2/3 activator, SCAR, and the *dock* mutation enhances a weak *SCAR* mutant phenotype during myoblast fusion, suggesting that Dock can promote SCAR-mediated Arp2/3 activation in some contexts (Kaipa et al., 2013). Although we have not been able to observe strong SCAR localization in either control or Dock-overexpressing egg chambers, it has been reported to localize to germline ring canals (Rodriguez-Mesa et al., 2012). However, *SCAR* germline mutants have a strong phenotype, which is more similar to that of Arp2/3 mutants (Hudson and Cooley, 2002; Zallen et al., 2002). Therefore, although we cannot rule out the possibility that WASp and SCAR may be partially redundant or capable of partially compensating for the loss of each other, because the *dock* mutant phenotype is milder than that observed for *SCAR*, it suggests that SCAR is not the primary Dock interactor in the germline.

### Dock and Msn may help to localize each other to ring canals and nurse cell membranes

In addition to the genetic interaction between Dock and the Arp2/3 complex, our data are consistent with Dock acting in a parallel pathway with Msn in the germline. Dock and Msn have been shown to interact biochemically and genetically in other contexts (Liu et al., 1999; Ruan et al., 1999; Su et al., 2000), but this is the first connection between the two in the egg chamber. To our knowledge, there is no evidence in flies or any other model system to suggest that Dock is a direct target of Msn, and the kinase activity of Msn is not required to promote ectopic enrichment of Dock at ring canals and nurse cell membranes (Fig. S2C). Therefore, it suggests that the connection between Msn and Dock could be through a localization dependency/hierarchy or a regulated physical interaction.

One possibility is that the connection between Dock and Msn in the germline involves a physical interaction, which then alters the activity of one or both proteins. During eye development in the fly, it has been proposed that Dock functions upstream of and negatively regulates Msn (Ruan et al., 1999), so this type of negative regulation could be occurring in the germline as well. For example, binding to Dock could hold Msn in an inactive conformation, preventing it from phosphorylating target substrates. Although Dock does not have any catalytic activity, if one or more SH3 domains can interact with multiple different proteins, interaction with Msn could prevent Dock from recruiting other proteins, such as WASp, to the germline ring canals. Such a competition between interactors has been demonstrated for the homolog Nck (Buvall et al., 2013). Additional work will be required to further investigate the genetic and physical interaction between these two in the germline.

In summary, we have characterized a novel role for a conserved adaptor protein in regulation of the size and stability of the germline intercellular bridges in the developing egg chamber. Dock is one of only a handful of proteins that localize to the mature intercellular bridges during gametogenesis in both males and females (Yamashita, 2018). Therefore, further dissecting the interactions between Dock, WASp, and Msn, as well as identifying other Dock-interacting proteins might provide important insight into the regulation of these essential structures during gametogenesis.

## MATERIALS AND METHODS

### Genetics

The GAL4/UAS system was used to regulate transgene expression in the female germline; the intensity of transgene expression can be modulated based on the temperature and length of time the flies are incubated prior to dissection (longer time points at higher temperatures increase transgene expression; Hudson and Cooley, 2014). Three different GAL4 lines were used. The maternal triple driver [*MTD-GAL4;* *otu-GAL4; nos-GAL4; nos-GAL4*; Bloomington *Drosophila* Stock Center (BDSC) #31777] shows relatively high GAL4 expression throughout oogenesis. The *Matαtub-GAL4* (BDSC #7063) driver begins to express GAL4 at around stage 2, and this expression persists through the rest of oogenesis. The *nanos-GAL4* (*nos-GAL4*) driver (BDSC #32563) expresses GAL4 in two pulses, the first in the germarium and the second beginning around stage 7 (Hudson and Cooley, 2014). In some experiments, the *GAL80^ts^* inhibitor was used in combination with *nos-GAL4* to control transgene expression (Kline et al., 2018; Thestrup et al., 2020).

The FLP/FRT system was used to create homozygous mutant germ cells in an otherwise heterozygous mutant background. To generate homozygous *dock^04723^FRT40/dock^04723^FRT40* mutant germline clones, the dominant female sterile mutation, *ovo^D1^ FRT40*, was used to select against egg chambers that had not undergone mitotic recombination. The presence of the *ovo^D1^* mutation will cause egg chambers to degenerate around stage 5, meaning the egg chambers that continue to develop past that stage are homozygous for the *dock* mutation (*dock^04723^FRT40/dock^04723^FRT40*) (Fig. 2B; Fig. S1A). To generate homozygous *dock^k13421^FRT40/ dock^k13421^FRT40* mutant nurse cells (Fig. S1B) in an otherwise heterozygous mutant background, *hs-FLP; ubi GFP FRT40* was crossed to *dock^k13421^FRT40/Cyo.* Adult female flies (*hs-FLP; dock^k13421^FRT40/ubi GFP FRT40*) were dissected and stained with an anti-HtsRC antibody (see below) and DAPI. Homozygous mutant cells (*dock^k13421^FRT40/ dock^k13421^FRT40*) lack GFP, whereas heterozygous (*dock^k13421^FRT40/ubi GFP* FRT40) and homozygous wild-type cells (*ubi GFP FRT40/ubi GFP FRT40*) will be marked by GFP. In this experiment, *hs-FLP; ubi GFP FRT40/Cyo* siblings were used as the control.

The following lines used in this study were obtained from the Bloomington *Drosophila* Stock Center: *w^1118^* (BDSC #3605), *UAS-msn-RNAi* (BDSC #42518), *UAS-arpC2-RNAi* (BDSC #43132), *dock^04723^/Cyo* (BDSC #11385, which was recombined on the FRT40 containing chromosome), *hsFLP;ovo^D1^ FRT40* (BDSC #2121), *UAS-src64-RNAi* (BDSC #36062), *WASp^1^/TM6B* (BDSC #51657), *arp3^515FC^ FRT80/TM3* (BDSC #39727), *msn^172^ FRT80B/TM6B* (BDSC #5947), *dock^k13421^* (BDSC #10444, which was recombined on the FRT40 containing chromosome). Additional fly lines include: *UAS-Myr-HA-MsnWT* (Kaneko et al., 2011), *UAS-Myr-HA-MsnKR* (Kline et al., 2018), and MsnYFP (DGGR #115454) (Kyoto Stock Center; Lowe et al., 2014; Lye et al., 2014; Rees et al., 2011), and *hs-FLP; ubi-GFP FRT40A*. For a complete list of the genotypes of all flies used in each experiment, refer to Table S1. Flies were maintained on standard cornmeal molasses medium at 25°C unless otherwise stated.

### Generation of novel Dock overexpression lines

For the *UAS-HA-Dock* and *UAS-Myr-HA-Dock* lines, the *dock* cDNA sequence (and ∼400 bp of the 3′UTR) corresponding to the longer isoforms (isoforms B, C and D) was amplified from the LD42588 cDNA clone (*Drosophila* Genomics Resource Center) and ligated into pENTR3c Dual (*Drosophila* Gateway; Carnegie Institute for Science) using In-Fusion HD Cloning (Takara Bio USA) and the primers indicated in Table S2. The Gateway LR Clonase II Enzyme Mix (ThermoFisher Scientific) was used to recombine the HA–Dock or Myr–HA–Dock sequences into the UASp-pPW germline expression vector (*Drosophila* Gateway, Carnegie Institute for Science). Plasmids were purified, and sequence verified before being injected into *w^1118^* flies; the resulting transgenic strains were balanced with either CyO (Chromosome II) or TM3, Sb (Chromosome III) (BestGene, Inc).

The remaining transgenic lines used in this study (*UASp-HA-Dock, UASp-HA-Dock SH3-1, UASp-HA-Dock SH3-2, UASp-HA-Dock SH3-3, UASp-HA-Dock SH3-1,2,3,* and *UASp-HA-Dock SH2, UASp-HA-Dock isoA*) were cloned into the YS041 pUASp attB vector (a gift from Lynn Cooley, Department of Genetics, Yale University School of Medicine, CT, USA), and the PhiC31 integrase was used to insert all transgenes into the same attP2 site (68A4 on chromosome III). Mutagenesis of the indicated domains was based on previously characterized mutants (Ruan et al., 1999; Rao and Zipursky, 1998). Mutagenesis was performed in the pENTR3C plasmid containing the HA–Dock–UTR sequence using a Q5 Mutagenesis kit (NEB) with the primers indicated in Table S2. In-Fusion HD Cloning (Takara Bio USA) was then used to transfer the mutated sequences from the pENTR3C plasmid to the YS041 pUASp attB vector (a gift from Lynn Cooley; Neelakanta et al., 2012). The phenotypes observed in egg chambers expressing the HA–Dock transgene from the attP2 site (YS041 plasmid) were weaker than in egg chambers expressing the HA–Dock transgene integrated by random p-element insertion (pPW plasmid). Therefore, we used the stronger *MTD-GAL4* (Figs 4 and 5A; Figs S2B, S3 and S4A) to express the various *UAS-HA-Dock* transgenes from the attP2 site throughout oognesis, and we used *Matαtub-GAL4* to express the *UAS-HA-Dock* or *UAS-Myr-HA-Dock* beginning at stage 2 for the lines generated through p-element insertion (Fig. 3; Fig. S2A,D).

### Dissections and staining

Female flies were placed in a vial of cornmeal molasses food containing fresh ground yeast and male flies. After 48–72 h incubation at either 25°C or 29°C, the flies were dissected in S2 medium (Genesee Scientific) under a stereomicroscope. Ovarian tissue was fixed with a 4% formaldehyde solution (in PBS) for 15 min and washed with PBS plus 0.1–1% Triton X-100. The tissue was stained with DAPI (1:500 dilution; 1 mg/ml stock, D3571 Thermo Fisher Scientific), phalloidin (1:500 dilution of TRITC or FITC; ECM Biosciences), and/or antibodies to various proteins of interest, including HtsRC (1:20; DSHB htsRC), Dock (1:400; Clemens et al., 1996), HA (1:200, Cat. #71-5500, Thermo Fisher Scientific), or WASp (DSHB P5E1; 1:10). Fluorescent secondary antibodies were used at a 1:200 dilution (anti-mouse-IgG conjugated to Alexa Fluor 488 or anti-rabbit-IgG conjugated to Alexa Fluor 555; Thermo Fisher Scientific or Jackson ImmunoResearch). All antibody dilutions were made in PBS plus 0.3–1% Triton X-100 and 5 mg/ml BSA. After antibody incubation, the tissue was washed with PBS plus 0.1–1% Triton X-100 and mounted using SlowFade antifade solution (ThermoFisher). Experimental conditions for each figure are summarized in Table S1.

### Imaging and analysis

Most images were collected using the 20× (0.4 NA HC Fl Plan) or 40× lens (0.65 NA HCX Fl Plan) without binning on a Leica DM5500 compound fluorescence microscope with a Leica DFC7000T camera with a motorized *z*-stage. Images for the analysis in Fig. S1B were collected using a Leica DMC4500 camera without binning. *Z*-stacks were acquired using Leica Application Suite X Software with the system optimized *z*-step. Some images (Fig. 4F; Fig. S4B) were deconvolved using the 3D Blind Deconvolution module within the LASX software (five total iterations). Images of the anti-Dock antibody stain in Fig. 1 were acquired using a Zeiss 710 confocal microscope with a 63× water lens (C-apochromat/1.2) controlled by Zen Black software. Egg chamber stages were determined using both morphology and egg chamber area based on average area ranges (Jia et al., 2016). Ring canals were counted or the diameters were measured from the HtsRC channels using the line tool in Fiji, and line scan analysis was performed as previously described (Kline et al., 2018; Thestrup et al., 2020). Data were graphed in Excel or Prism 9 (GraphPad). Statistical significance was determined using the Kolmogorov–Smirnov test, unpaired two-tailed *t*-test, or one-way ANOVA with Tukey's multiple comparison (*P*<0.05) (Prism 9; GraphPad).

### Western blot analysis

Ovaries were dissected in S2 medium and processed as described previously (Kline et al., 2018). The ovaries were mechanically disrupted using a pestle in 60 µl of homogenization buffer [83 mM Tris-HCl pH 6.8, 2.7% SDS, 189 mM β-mercaptoethanol, 1× Proteoguard protease inhibitor (Clontech Proteoguard, 100× stock), and molecular grade water]. Samples were incubated at 100°C for 10 min before being spun at 18,800 ***g*** for 5 min at room temperature. For the data in Fig. 4B, the supernatant (3 µl) was added to a fresh tube with 17 µl of 4× Laemmli sample buffer, all of which was then loaded onto a 4–20% gradient gel (Bio-Rad). For the data in Fig. S2A, 60 µl of supernatant from the spin was diluted with 20 µl of 4× Laemmli sample buffer. A total of 20 µl was loaded in the first set of lines, and 18 µl of a 1:4 dilution was loaded in the second set of lanes. Proteins were transferred onto a Trans-Blot membrane (BioRad). After blocking [TBST (TBS containing 0.1% Tween 20) plus 5% milk], the membrane was incubated in primary antibody solution [1:100 anti-Tubulin (DSHB E7), 1:250 anti-HA (Cat. #71-5500, Thermo Fisher Scientific), or anti-Dock (1:10,000; Clemens et al., 1996)] made in TBST plus 5% milk. After washing, the secondary solution – 1:600 anti-mouse-IgG conjugated to DyLight 650 (Thermo Fisher Scientific #84545) and 1:5000 anti-rabbit-IgG conjugated to HRP (GE Healthcare) in TBST plus 5% milk – was incubated with rocking at room temperature. HA-tagged proteins or Dock were visualized using the Clarity Max Western ECL Luminol enhancer solution (BioRad); the membrane was imaged using a FluroChem Q (Protein Simple)*.* Band density was measured using the FluroChemQ software. The tubulin bands were used as the loading control. Values show the relative levels of either HA (with the intensity for the HA–Dock band set to 1; Fig. 4B), or Dock protein (with the intensity for the control set to 1.0; Fig. S2A).

### Mature egg imaging and viability assays

Female flies of the appropriate genotype were incubated with ground yeast in the presence of males for ∼24 h before being transferred to apple juice plates with wet yeast. The apple juice plates were replaced every ∼24 h. Mature eggs were collected from the apple juice plates and imaged using a Zeiss-LP520 microscope with a ProgRes MF camera (Jenoptik) using ProgRes Capture Pro Software (Jenoptik). The lengths and widths of the eggs were determined using the line tool in Fiji/ImageJ. The volume was calculated using the equation volume=1/6π(length)(width)^2^. Embryonic viability was assessed by placing mature eggs on fresh apple juice plates and recording the percentage that has hatched after >24 h at 25°C.

## Supplementary Material

Supplementary information

Reviewer comments
