# Supplementary material for: Precise levels of the Drosophila adaptor protein Dreadlocks maintain the size and stability of germline ring canals
Source: J Cell Sci. 2021 Apr 27;134(8):jcs254730. doi: 10.1242/jcs.254730 (PMC8106954; doi:10.1242/jcs.254730)
Supplement: Supplementary information [file joces-134-254730-s1.pdf]

Stark et al. Figure S1

A

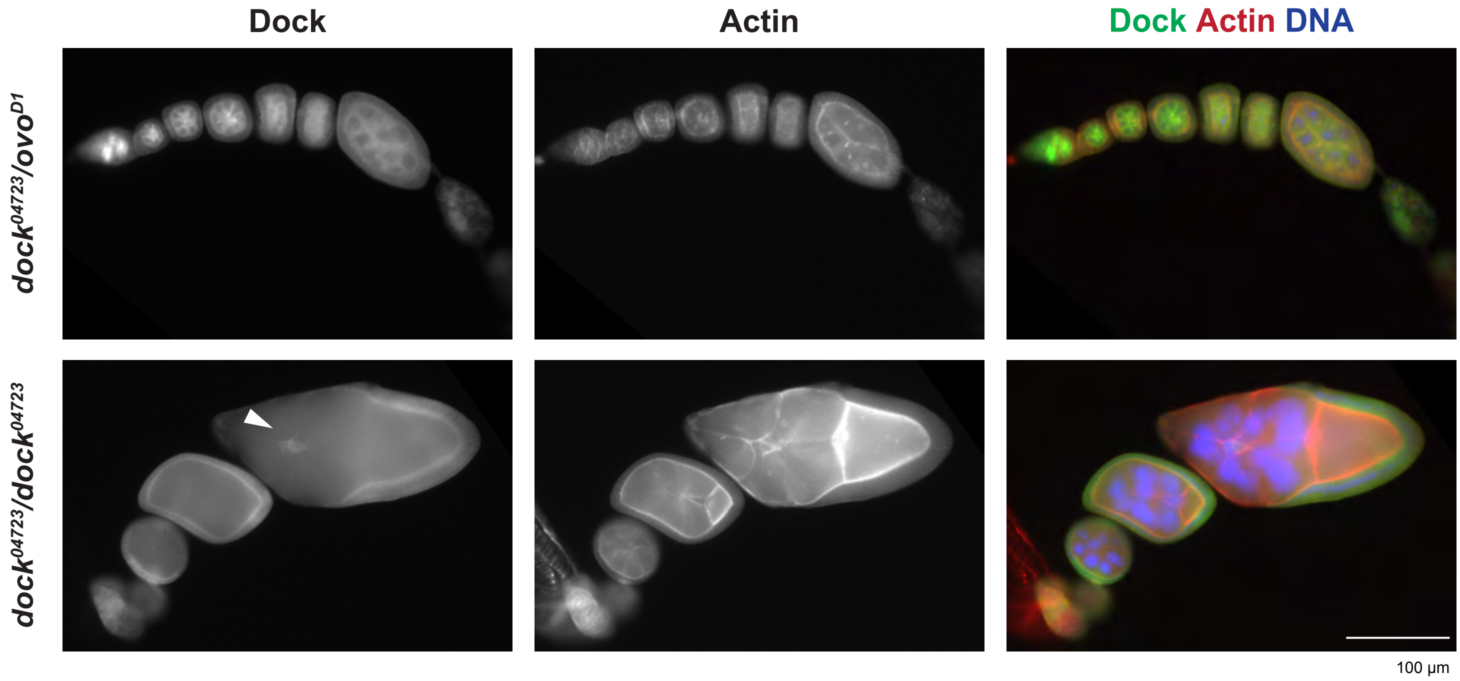

B

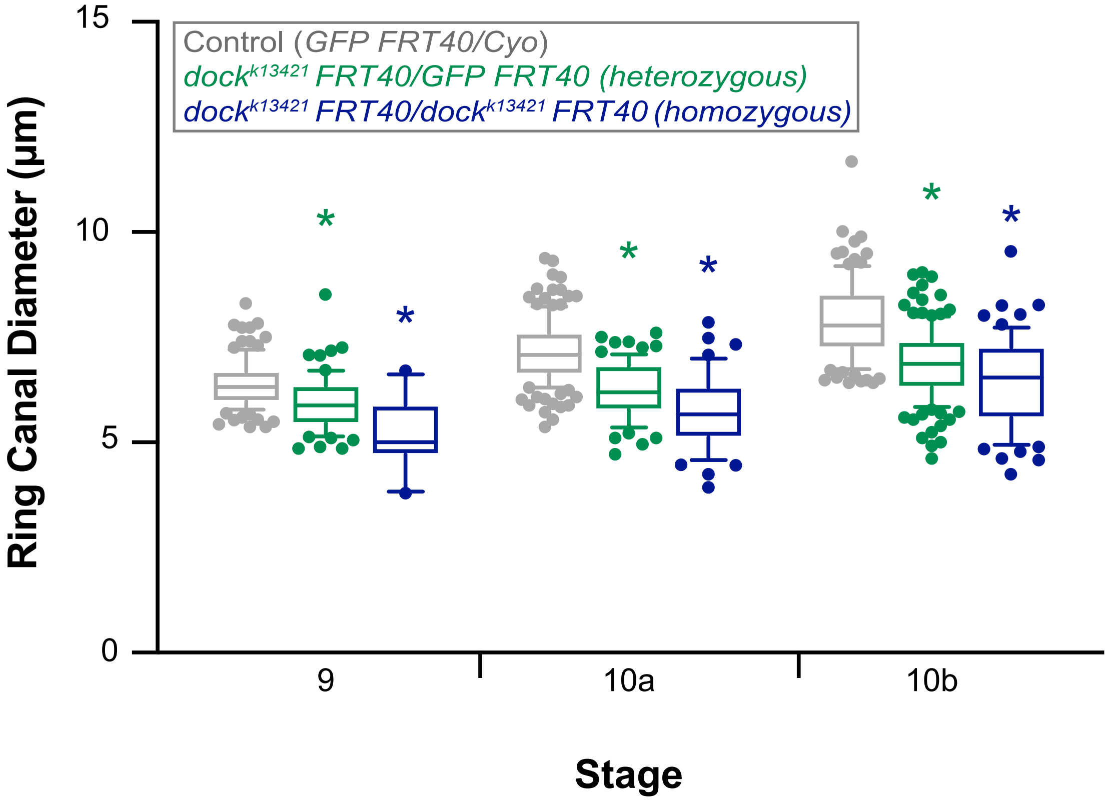

Journal of Cell Science • Supplementary information

Fig. S1: Specificity of the Dock antibody and *dock^{k13421}* ring canal phenotype. (A) *dock^{04723} FRT40/ ovo^{D1} FRT40* or *dock^{04723}FRT40/ dock^{04723} FRT40* egg chambers stained with the anti-Dock antibody. Egg chambers containing nurse cells homozygous for the Dock mutation (*dock^{04723} FRT40/ dock^{04723} FRT40*) were generated using the DFS-FLP/FRT method. The *dock^{04723} FRT40/ ovo^{D1} FRT40* egg chambers show Dock localization in the germline within the germarium and younger stages before the egg chambers begin to degenerate due to the presence of the dominant *ovo^{D1}* mutation. The *dock^{04723}FRT40/ dock^{04723} FRT40* egg chambers show a significant reduction in Dock staining in the germline, but Dock protein is still present in the somatic follicle cells and the border cell cluster (arrowhead) that is migrating through the center of the germ cells in the stage 9 egg chamber. (B) Box and whiskers plot showing the diameter of ring canals connecting nurse cells with the following genotypes: *ubi GFP FRT40/Cyo*, *dock^{k13421}FRT40/ubi GFP FRT40* (heterozygotes), and *dock^{k13421}FRT40/ dock^{k13421}FRT40* (homozygous mutant). The box represents the 25-75th percentiles, and the median is indicated. The whiskers show the 10-90th percentiles. Individual points represent values outside of that range. n= 11-143 ring canals/stage/condition. Asterisks indicate significant difference compared to both other conditions at that stage ($p<0.05$, one-way ANOVA with Tukey's multiple comparison post-hoc)

Journal of Cell Science • Supplementary information

Stark et al. Figure S2

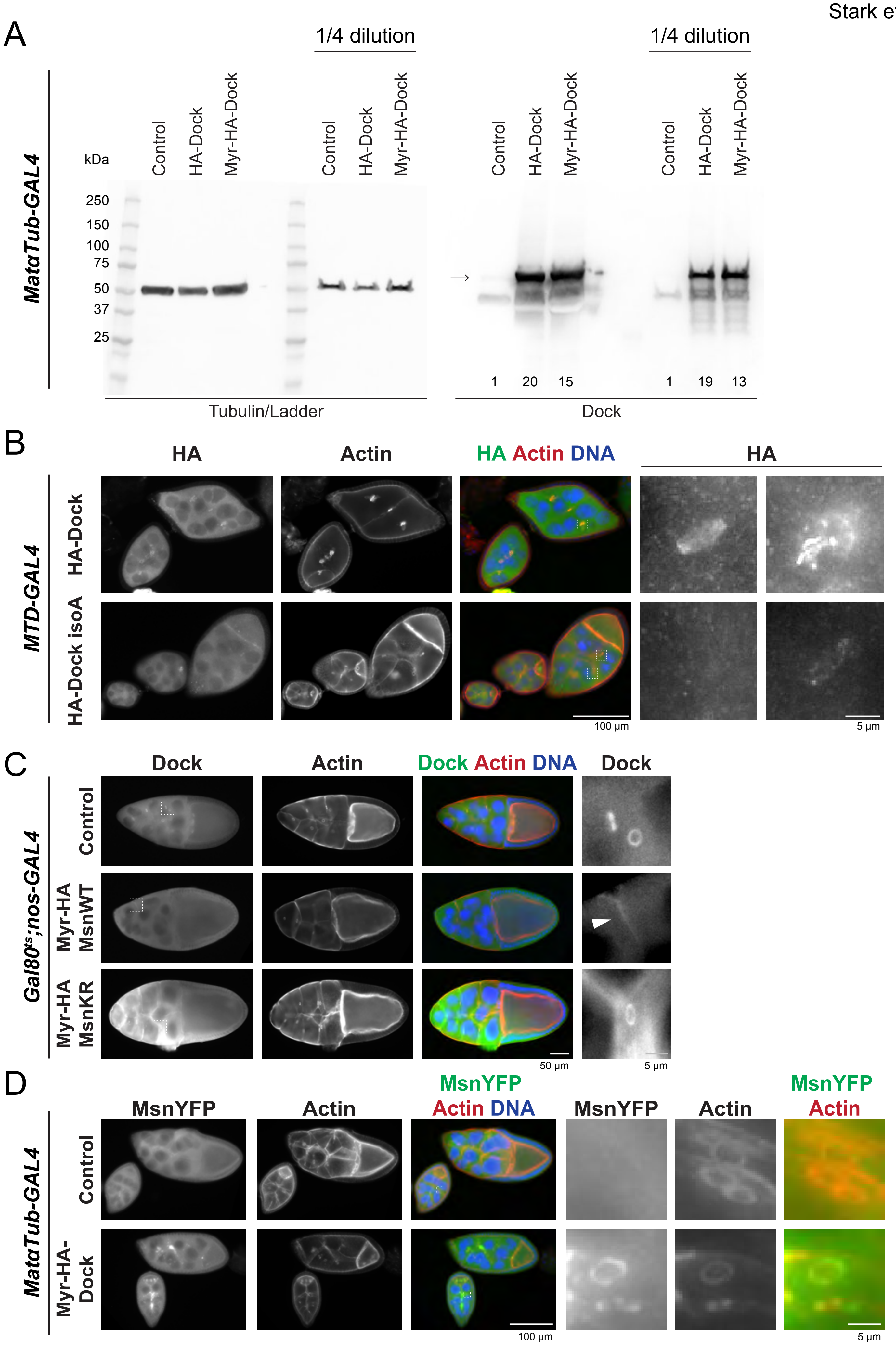

Journal of Cell Science • Supplementary information

Fig. S2: Dock over-expression and ability of Dock and Msn to recruit each other in the germline. (A) Western blot of whole ovary lysate from control, HA-Dock, or Myr-HA-Dock expressing tissue. Tubulin was used as the loading control, and values indicate relative expression level of Dock (long isoform indicated by arrow) in the HA-Dock or Myr-HA-Dock; expression level of Dock in the control was set to 1.0. (B) Because there is evidence that a shorter ~45 kDa isoform of Dock (isoA) may be expressed in the ovary, we tested over-expression of that short isoform in the germline. Compared to expression of HA-Dock (long isoform), expression of HA-Dock isoA in the germline caused a much milder phenotype. Further, we did not detect strong localization of HA-Dock isoA at germline ring canals. (C) Fluorescence images of control, Myr-HA-MsnWT, and Myr-HA-MsnKR expressing stage 10 egg chambers. Because the Dock staining was so much brighter in the Myr-HA-MsnKR expressing egg chambers, these panels were not scaled equivalently to the control and Myr-HA-MsnWT expressing egg chambers. Arrowhead points to nurse cell membrane. (D) Maximum intensity projections of fluorescence images of control and Myr-HA-Dock expressing egg chambers which also express an endogenously-tagged MsnYFP. Boxes (B,C,D) indicate regions shown in the panels to the right.

Journal of Cell Science • Supplementary information

Stark et al. Figure S3

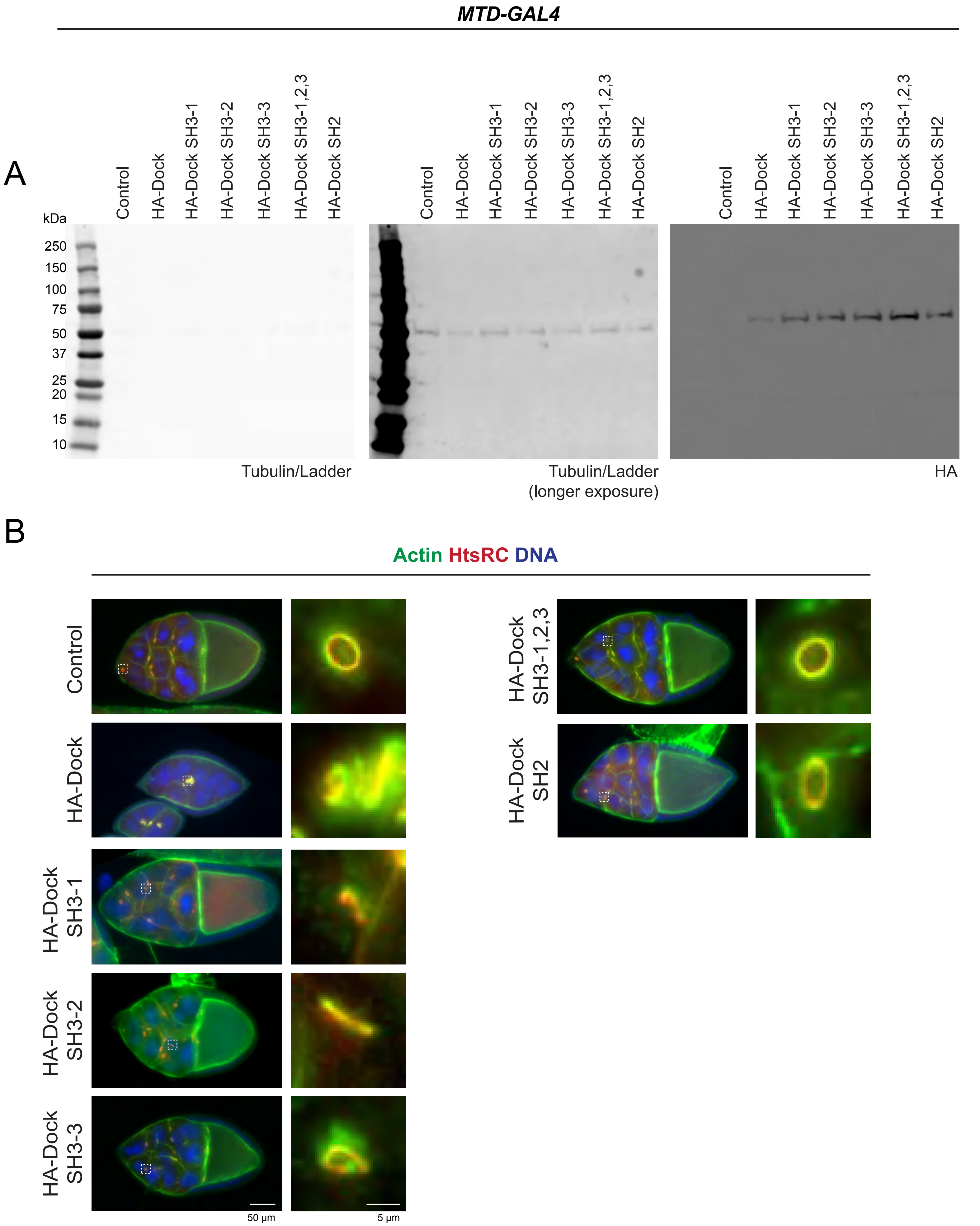

Journal of Cell Science • Supplementary information

Fig. S3: HA-Dock transgenes are expressed at similar levels but have different effects when expressed in the germline. (A) Full size blot for the experiment shown in Fig. 4B. Western blot of whole ovary lysate from control or HA-Dock expressing tissue. Tubulin was used as the loading control. Flies were incubated for 48 hours at 29°C prior to dissection. *MTD-GAL4* was used. (B) Fluorescence images of control and HA-Dock expressing egg chambers stained with an anti-HtsRC antibody, phalloidin, and DAPI. *MTD-GAL4* was used, and flies were incubated for 72 hours at 29°C prior to dissection. Images were scaled to allow the best visualization of germ cell structures.

Journal of Cell Science • Supplementary information

Stark et al. Figure S4

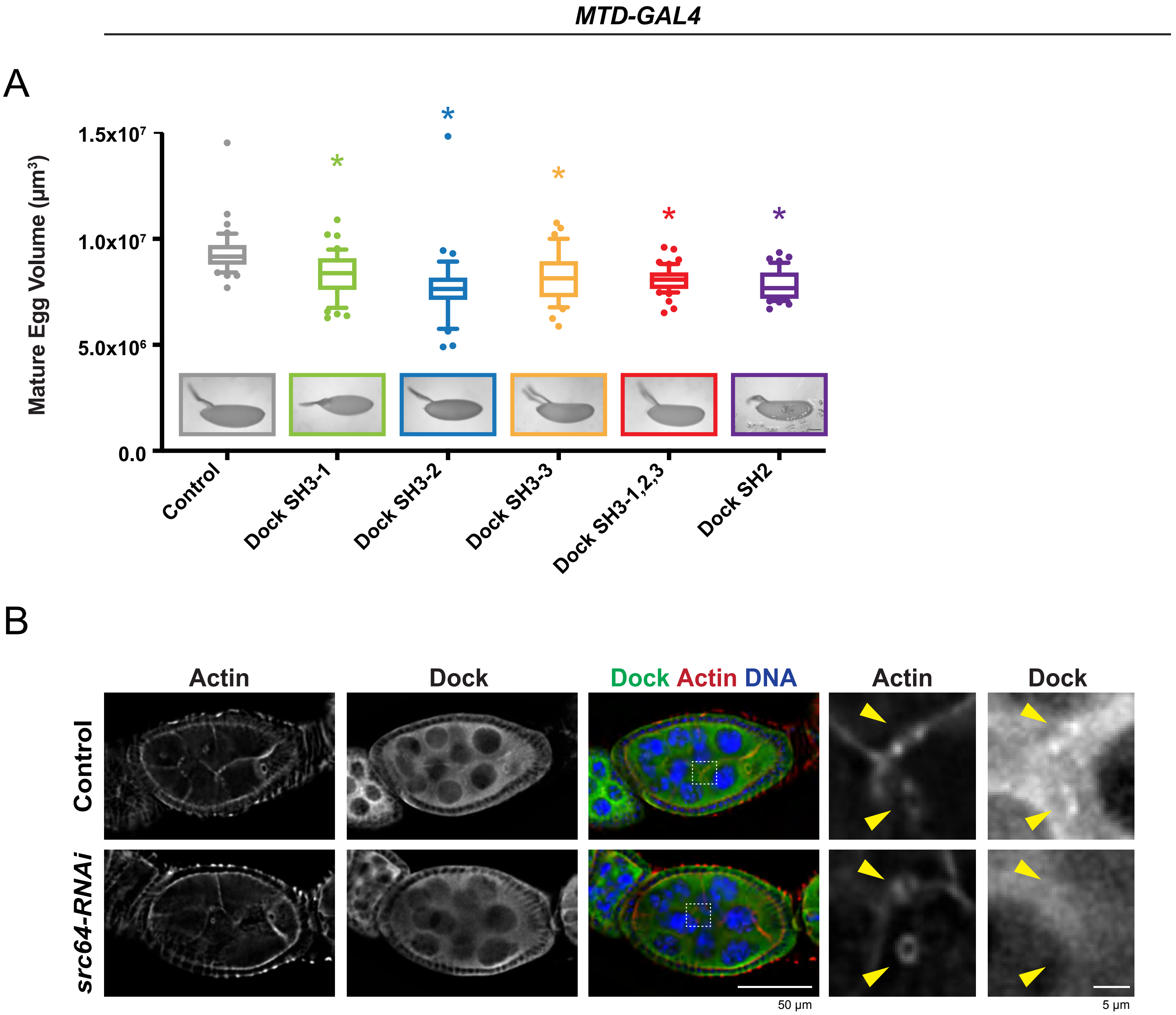

Fig. S4: Expression of HA-Dock alters mature egg volume, and depletion of Src64 reduces the localization of Dock to germline ring canals. (A) Box and whiskers plot showing the volume of mature eggs from control, HA-Dock SH3-1, HA-Dock SH3-2, HA-Dock SH3-3, HA-Dock SH3-1,2,3, or HA-Dock SH2 expressing egg chambers. No mature eggs were ever collected from the HA-Dock expressing egg chambers. The box represents the 25-75th percentiles, and the median is indicated. The whiskers show the 10-90th percentiles. Individual points represent values outside of that range. n=38-50 mature eggs per condition. Asterisks indicate a significant difference compared to control ($p<0.05$, one-way ANOVA with Tukey's multiple comparison post-hoc). Representative images of mature eggs for each condition are shown. Scale bar is 100 µm. (B) Control and *src64-RNAi* egg chambers at stage 6 stained with an anti-Dock antibody, phalloidin, and DAPI. *MTD-GAL4* was used, and flies were incubated at 29°C for 70 hours prior to dissection. Arrowheads indicate the position of the ring canals.

Journal of Cell Science • Supplementary information

Stark et al. Figure S5

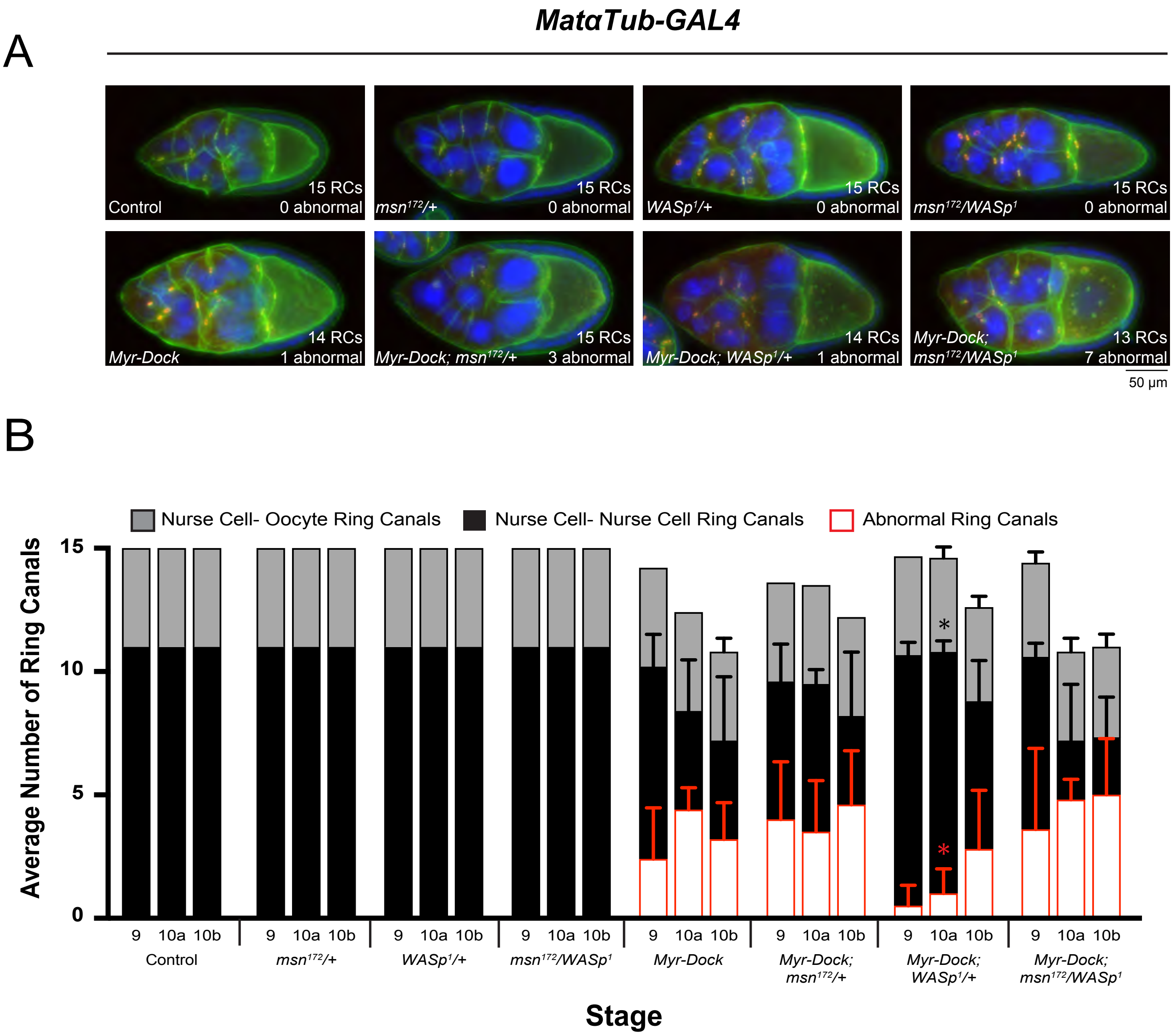

Figure S5: A heterozygous mutation in *msn* either alone or in combination with a mutation in *WASp* did not provide a strong rescue of the Myr-Dock expression phenotype. (A) Maximum intensity projections of fluorescence images of a late stage 9/early stage 10a control egg chamber and egg chambers expressing Myr-HA-Dock with *matαTub-Gal4* alone or in combination with the heterozygous mutations, $WASp^1/+$ and/or $msn^{172}/+$. Flies were incubated at 29°C for 45 hours prior to dissection, and tissue was stained with an anti-HtsRC antibody, phalloidin, and DAPI. (B) Average number of visible ring canals connecting nurse cells and nurse cells to the oocyte in the conditions shown in (A). Graph also shows the average number of "abnormal" (collapsed, lumen-less, or detached) ring canals, all of which were originally connecting nurse cells. Error bars represent standard deviation. n=4-6 egg chambers/stage/condition.

Journal of Cell Science • Supplementary information

Table S1: Genotypes and conditions for each experiment. The genotype of dissected flies used for each experiment, incubation temperatures and timepoints, and antibodies used are indicated.

Figure	Genotype	Temperature cross raised at (°C)	Incubation temperature	Incubation Time	Primary antibodies
**1A-C**	*w[1118]/otu-GAL4; nos-GAL4/+; nos-GAL4/+*	25	29	68	α-Dock
**1D**	*otu-GAL4/+; dock[04723] FRT40/nos-GAL4; UAS-dock-RNAi/nos-GAL4*				
**2A,B,D,E**	*w[1118]/otu-GAL4; nos-GAL4/+; nos-GAL4/+*	25	29	71	α-HtsRC
	otu-GAL4/+; dock[04723] FRT40/nos-GAL4; UAS-dock-RNAi/nos-GAL4				
	hsFLP; dock[04723] FRT40/dock[04723] FRT40				
**2C**	*w[1118]/otu-GAL4; nos-GAL4/+; nos-GAL4/+*	25	29	64	
	otu-GAL4/+; dock[04723] FRT40/nos-GAL4; UAS-dock-RNAi/nos-GAL4				
**3A**	*w[1118]/+;; matatub-GAL4/+*	25	29	43	α-HtsRC
	UASp-HA-Dock/+; matatub-GAL4/+				
	UASp-Myr-HA-Dock/+; matatub-GAL4/+				
**3B**	*w[1118]/+;; matatub-GAL4/+*	25	29	44	α-HA
	UASp-HA-Dock/+; matatub-GAL4/+				
	UASp-Myr-HA-Dock/+; matatub-GAL4/+				
**4B**	*w[1118]/otu-GAL4; nos-GAL4/+; nos-GAL4/+*	25	29	48	α-HA and α-Tubulin
	otu-GAL4/+; nos-GAL4/+; UASp-HA-Dock/nos-GAL4				
	otu-GAL4/+; nos-GAL4/+; UASp-HA-Dock-SH3-1/nos-GAL4				
	otu-GAL4/+; nos-GAL4/+; UASp-HA-Dock-SH3-2/nos-GAL4				
	otu-GAL4/+; nos-GAL4/+; UASp-HA-Dock-SH3-3/nos-GAL4				
	otu-GAL4/+; nos-GAL4/+; UASp-HA-Dock-SH3-1,2,3/nos-GAL4				
	otu-GAL4/+; nos-GAL4/+; UASp-HA-Dock-SH2/nos-GAL4				
**4C**	*w[1118]/otu-GAL4; nos-GAL4/+; nos-GAL4/+*	25	29	72	α-HA
	otu-GAL4/+; nos-GAL4/+; UASp-HA-Dock-WT/nos-GAL4				
	otu-GAL4/+; nos-GAL4/+; UASp-HA-Dock-SH3-1/nos-GAL4				
	otu-GAL4/+; nos-GAL4/+; UASp-HA-Dock-SH3-2/nos-GAL4				
	otu-GAL4/+; nos-GAL4/+; UASp-HA-Dock-SH3-3/nos-GAL4				
	otu-GAL4/+; nos-GAL4/+; UASp-HA-Dock-SH3-1,2,3/nos-GAL4				
	otu-GAL4/+; nos-GAL4/+; UASp-HA-Dock-SH2/nos-GAL4				
**4D,E**	*w[1118]/otu-GAL4; nos-GAL4/+; nos-GAL4/+*	25	29	72	α-HtsRC
	otu-GAL4/+; nos-GAL4/+; UASp-HA-Dock-SH3-1/nos-GAL4				
	otu-GAL4/+; nos-GAL4/+; UASp-HA-Dock-SH3-2/nos-GAL4				
	otu-GAL4/+; nos-GAL4/+; UASp-HA-Dock-SH3-3/nos-GAL4				
	otu-GAL4/+; nos-GAL4/+; UASp-HA-Dock-SH3-1,2,3/nos-GAL4				
	otu-GAL4/+; nos-GAL4/+; UASp-HA-Dock-SH2/nos-GAL4				
**4F**	*w[1118]/otu-GAL4; nos-GAL4/+; nos-GAL4/+*	25	29	70	α-Dock
	w[1118]/otu-GAL4; nos-GAL4/+; nos-GAL4/UAS-Src64-RNAi				

Journal of Cell Science • Supplementary information

**5A**	*w 1118/otu-GAL4; nos-GAL4/+; nos-GAL4/+*	25	29	48	α-WASp
	otu-GAL4/+; nos-GAL4/+; UASp-HA-Dock/nos-GAL4				
	otu-GAL4/+; nos-GAL4/+; UASp-HA-Dock-SH3-1/nos-GAL4				
	otu-GAL4/+; nos-GAL4/+; UASp-HA-Dock-SH3-3/nos-GAL4				
	otu-GAL4/+; nos-GAL4/+; UASp-HA-Dock-SH2/nos-GAL4				
**5B-D**	*w 1118/+;; matatub-GAL4/+*	25	29	44	α-HtsRC
	WASp 1/matatub-Gal4				
	UASp-Myr-HA-Dock/+; matatub-Gal4/+				
	UASp-Myr-Dock; WASp 1/ matatub-Gal4				
**6**	*w 1118/+;; matatub-GAL4/+*	25	29	44	α-HtsRC
	arp3 515FC/matatub-Gal4				
	UASp-Myr-HA-Dock/+; matatub-Gal4/+				
	UASp-Myr-Dock; arp3 515FC/ matatub-Gal4				
**7A,B**	*w 1118/+;; nos-Gal4/+*	25	29	71	α-HtsRC
	dock 04723/+; nos-Gal4/+				
	arpC2-RNAi/nos-Gal4				
	dock 04723/+; arpC2-RNAi/ nos-Gal4				
	msn-RNAi/+; nos-Gal4/+				
	dock 04723/msn-RNAi; nos-Gal4/+				
**7C**	*w 1118/+;; nos-Gal4/+*	25	29	71	
	dock 04723/+; nos-Gal4/+				
	arpC2-RNAi/nos-Gal4				
	dock 04723/+; arpC2-RNAi/ nos-Gal4				
	msn-RNAi/+; nos-Gal4/+				
	dock 04723/msn-RNAi; nos-Gal4/+				

Supplementary Figures

Figure	Genotype	Temperature cross raised at (°C)	Incubation temperature	Incubation Time	Primary antibodies
**S1A**	*hsFLP; dock 04723 FRT40/ovo D1 FRT40*	25	25	48-72	α-Dock
	hsFLP; dock 04723 FRT40/dock 04723 FRT40				
**S1B**	*hsFLP; ubiGFP FRT40/Cyo*				
	hsFLP; dock k13421 FRT40/ubiGFP FRT40				
**S2A**	*w 1118/+;; matatub-GAL4/+*	25	29	43	α-Dock and α-Tubulin
	UASp-HA-Dock/+; matatub-GAL4/+				
	UASp-Myr-HA-Dock/+; matatub-GAL4/+				
**S2B**	*otu-GAL4/+; nos-GAL4/+; UASp-HA-Dock/nos-GAL4*	25	29	48	α-HA

Journal of Cell Science • Supplementary information

	otu-GAL4/+; nos-GAL4/+; UASp-HA-Dock-isoA/nos-GAL4				
**S2C**	*w ^{1118}/+;Gal80ts/+; nos-GAL4/+*	18	29	44	α-Dock
	w ^{1118}/+;Gal80ts/+; nos-GAL4/UAS-Myr-HA-MsnWT				
	w ^{1118}/+;Gal80ts/UAS-Myr-Msn-MsnKR; nos-GAL4/+				
**S2D**	*w ^{1118}/+;; matatub-GAL4, MsnYFP/MsnYFP*	25	29	45	α-GFP
	w ^{1118}/+;UASp-Myr-HA-Dock/+; matatub-GAL4, MsnYFP/MsnYFP				
**S3A**	*w ^{1118}/otu-GAL4; nos-GAL4/+; nos-GAL4/+*	25	29	48	α-HA and α-Tubulin
	otu-GAL4/+; nos-GAL4/+; UASp-HA-Dock/nos-GAL4				
	otu-GAL4/+; nos-GAL4/+; UASp-HA-Dock-SH3-1/nos-GAL4				
	otu-GAL4/+; nos-GAL4/+; UASp-HA-Dock-SH3-2/nos-GAL4				
	otu-GAL4/+; nos-GAL4/+; UASp-HA-Dock-SH3-3/nos-GAL4				
	otu-GAL4/+; nos-GAL4/+; UASp-HA-Dock-SH3-1,2,3/nos-GAL4				
	otu-GAL4/+; nos-GAL4/+; UASp-HA-Dock-SH2/nos-GAL4				
**S3B**	*w ^{1118}/otu-GAL4; nos-GAL4/+; nos-GAL4/+*	25	29	72	α-HtsRC
	otu-GAL4/+; nos-GAL4/+; UASp-HA-Dock/nos-GAL4				
	otu-GAL4/+; nos-GAL4/+; UASp-HA-Dock-SH3-1/nos-GAL4				
	otu-GAL4/+; nos-GAL4/+; UASp-HA-Dock-SH3-2/nos-GAL4				
	otu-GAL4/+; nos-GAL4/+; UASp-HA-Dock-SH3-3/nos-GAL4				
	otu-GAL4/+; nos-GAL4/+; UASp-HA-Dock-SH3-1,2,3/nos-GAL4				
	otu-GAL4/+; nos-GAL4/+; UASp-HA-Dock-SH2/nos-GAL4				
**S4A**	*w ^{1118}/otu-GAL4; nos-GAL4/+; nos-GAL4/+*	25	29	72	
	otu-GAL4/+; nos-GAL4/+; UASp-HA-Dock-SH3-1/nos-GAL4				
	otu-GAL4/+; nos-GAL4/+; UASp-HA-Dock-SH3-2/nos-GAL4				
	otu-GAL4/+; nos-GAL4/+; UASp-HA-Dock-SH3-3/nos-GAL4				
	otu-GAL4/+; nos-GAL4/+; UASp-HA-Dock-SH3-1,2,3/nos-GAL4				
	otu-GAL4/+; nos-GAL4/+; UASp-HA-Dock-SH2/nos-GAL4				
**S4B**	*w ^{1118}/otu-GAL4; nos-GAL4/+; nos-GAL4/+*	25	29	70	α-Dock
	w ^{1118}/otu-GAL4; nos-GAL4/+; nos-GAL4/UAS-Src64-RNAi				
**S5**	*w ^{1118}/+;; matatub-GAL4/+*	25	29	45	α-HtsRC
	msn ^{172}/matatub-Gal4				
	WASp ^{1}/matatub-Gal4				
	WASp ^{1}/ msn172, mataTub-Gal4				
	UASp-Myr-HA-Dock/+; matatub-Gal4/+				
	UASp-Myr-Dock; msn ^{172}/ matatub-Gal4				
	UASp-Myr-Dock; WASp1/ matatub-Gal4				
	UASp-Myr-Dock/+;WASp ^{1}/ msn172, mataTub-Gal4				

Journal of Cell Science • Supplementary information

Table S2: Primers used to generate transgenic lines. The primers and vectors used to generate novel transgenic lines or induce mutations are indicated.

Purpose	Destination Vector	Forward Primer	Reverse primer
Generate Myr-HA-Dock with ~400 bp of 3'UTR		ATGGGTAATTGTTTAACTTATCCGTATGATGTTCCTGATTATGCATACCCCTACGACGTGCCAGACTACGCGGGT	ACGGTATGTAAATATGTAATGTATTGCATGTTGACA
Generate HA-Dock with ~400 bp of 3'UTR		ATGTATCCGTATGATGTTCCTGATTATGCATACCCCTACGACGTGCCAGACTACGCGGGTATGTTGGAACACCCCCAGCGGTTT	ACGGTATGTAAATATGTAATGTATTGCATGTTGACA
Clone Myr-HA-Dock	pENTR3C	TCAGTCGACTGGATCCATGGGTAATTGTTTAACTTATCCGTATGA	GTCTAGATATCTCGAGACGGTATGTAAATATGTAATGTATTGCATG
Clone HA-Dock	pENTR3C	TCAGTCGACTGGATCCATGTATCCGTATGATGTTCCTGATTATG	GTCTAGATATCTCGAGACGGTATGTAAATATGTAATGTATTGCATG
Clone long Dock isoforms	YS041	TAGTGGATCTGGATCCATGTATCCGTATGATGTTCCTGATTATGCA	CGAGGTCGACTCTAGAACGGTATGTAAATATGTAATGTATTGCATGTTGAC
Clone short Dock isoform A	YS041	TAGTGGATCTGGATCCATGTATCCGTATGATGTTCCTGATTATGCA	CGAGGTCGACTCTAGAACGGTATGTAAATATGTAATGTATTGCATGTTGAC
Primer to add HA tag to Dock isform A		ATGTATCCGTATGATGTTCCTGATTATGCATACCCCTACGACGTGCCAGACTACGCGATGGCGGGCAACATGAAGCA	

UAS-Dock line	Mutation	Forward Primer	Reverse primer
SH2	R474Q	CTTCCTCATCCAAGACAGTGAGACTAACATGGGAGAC	TCGCCATCGTGCCCGTGG
SH3-1	W186K	CTCCAAGCACAAATGGCGCGTCCAGAAC	TCGTCCAGCAGCAGGTAG
SH3-2	W289K	CAACGATGGAAAGTGGCGTGGAC	GACTTCTCCAGGATGAGG
SH3-3	W363K	CGATCCGGACAAATACAAAGCTCGCAACAATCAGGGCC	GAGGCGGGCCGATCCACG

Journal of Cell Science • Supplementary information
