# Supplementary material for: Precise levels of the Drosophila adaptor protein Dreadlocks maintain the size and stability of germline ring canals
Source: J Cell Sci. 2021 Apr 27;134(8):jcs254730. doi: 10.1242/jcs.254730 (PMC8106954; doi:10.1242/jcs.254730)
Supplement: Reviewer comments [file joces-134-254730_review_history.pdf]

Precise levels of the *Drosophila* adaptor protein Dreadlocks maintain the size and stability of germline ring canals

Kara Stark, Olivia Crowe and Lindsay Lewellyn
DOI: 10.1242/jcs.254730

Editor: David Glover

Review timeline

Original submission:	21 September 2020
Editorial decision:	9 November 2020
First revision received:	10 February 2021
Accepted:	15 March 2021

Original submission

First decision letter

MS ID#: JOCES/2020/254730

MS TITLE: Precise levels of the adaptor protein, Dreadlocks, maintain the size and stability of germline ring canals in the developing egg chamber

AUTHORS: Kara Stark, Olivia Crowe, and Lindsay Lewellyn
ARTICLE TYPE: Research Article

We have now reached a decision on the above manuscript.

To see the reviewers' reports and a copy of this decision letter, please go to: https://submit-jcs.biologists.org and click on the 'Manuscripts with Decisions' queue in the Author Area. (Corresponding author only has access to reviews.)

As you will see, the reviewers raise a number of substantial criticisms that prevent me from accepting the paper at this stage. They suggest, however, that a revised version might prove acceptable, if you can address their concerns. If you think that you can deal satisfactorily with the criticisms on revision, I would be pleased to see a revised manuscript. We would then return it to the reviewers.

We are aware that you may be experiencing disruption to the normal running of your lab that makes experimental revisions challenging. If it would be helpful, we encourage you to contact us to discuss your revision in greater detail. Please send us a point-by-point response indicating where you are able to address concerns raised (either experimentally or by changes to the text) and where you will not be able to do so within the normal timeframe of a revision. We will then provide further guidance. Please also note that we are happy to extend revision timeframes as necessary.

Please ensure that you clearly highlight all changes made in the revised manuscript. Please avoid using 'Tracked changes' in Word files as these are lost in PDF conversion.

I should be grateful if you would also provide a point-by-point response detailing how you have dealt with the points raised by the reviewers in the 'Response to Reviewers' box. Please attend to all of the reviewers' comments. If you do not agree with any of their criticisms or suggestions please explain clearly why this is so.

© 2021. Published by The Company of Biologists under the terms of the Creative Commons Attribution License (https://creativecommons.org/licenses/by/4.0/).

Reviewer 1

Advance summary and potential significance to field

This manuscript, by Stark et al., uses *Drosophila* oocytes to examine the role of *dreadlock* (dock), the fly homolog of NCK adaptor protein, in ring canal formation. Dock contains one SH2 and 3 SH3 domains, and is known to have a role in axonal pathfinding and interact with actin regulators. As the role of Dock in ring canals has not been investigated, the authors first show that Dock proteins associate with ring canals since early stages of oogenesis. The localization of Dock at the ring canals depends on its SH2 domain and the Src tyrosine kinase, suggesting this association is mediated by the binding of Dock SH2 domain to a phosphotyrosine-containing Src substrate. Loss of *dock* function, by either RNAi or a loss-of-function mutation, leads to alterations in the size of ring canals and reductions in the levels of actin and HtsRC at ring canals. On the other hand, overexpression of Dock in female germ cells causes severe malformation of ring canals, and this phenotype is abolished when its SH2 domain or all three SH3 domains are mutated. *WASp* and *arp*3 dominantly suppress Dock overexpression phenotype, and Dock overexpression increases the presence of WASp proteins in the vicinity of ring canals, suggesting that ectopic Dock recruits WASp to affect ring canal morphology.
Consistent with this, reduction of *arpC2*, a component of actin nucleation Arp2/3 complex, suppresses Dock overexpression. Conversely, reduction of *dock* enhances the ring canal phenotype of *arpC2*^{RNAi}. Taken together, the authors propose that Dock, recruited to the ring canals by Src64-mediated phosphorylation, regulates ring canal morphology by facilitating Arp2/3-dependent actin nucleation.

Comments for the author

As the ring canals regulate the passage of materials between adjacent cells and are critical for germ cell development, a better understanding of their regulation is certainly of interests to cell biologists. This manuscript is well written and the findings are well presented, although some of the conclusions are not supported by the data. Thus, this manuscript needs to be revised before it is appropriate for publication in JCS.

Major issues:

1. Compared to the *dock* RNAi-knockdowned oocytes, *dock* germline clones showed a stronger and more consistent reduction in ring canal size. The authors explain this discrepancy by demonstrating the presence of residual Dock proteins in the RNAied oocytes. However, another possibility is that the *FRT, dock*^{04723} chromosome contains a secondary mutation, which causes ring canal size reduction. Given the importance of *dock* loss-of-function phenotype, this should be addressed by additional experiments. The fact that *dock* overexpression has severe oogenesis phenotype may complicate a rescue experiment. How about using a different *dock* allele to rule out the possibility of background mutation in the *dock*^{04723}?
2. Oocytes overexpressing *dock* exhibited severely disrupted ring canals, which is not the opposite of the loss-of-function phenotype (*dock* germline clones showed smaller ring canals, but unaffected ring canal morphology and composition). Even in the *myr-dock* overexpression, which has milder defects, it is not clear that the ring canal sizes are affected. The authors put forth an explanation that the *dock* protein level needs to be tightly regulated to control ring canal size. Given the importance of Dock protein level, is it possible to estimate the level of HA-Dock overexpression in *Mat Tub>HA-Dock* relative to endogenous Dock?
3. The phenotypic similarities between Dock overexpression and myr-Msn suggest these two genes interact in ring canal formation. Consistent with this, the authors provide evidence that Msn and Dock recruit each other to nurse cell membrane. However, the genetic interaction data between the two were negative. Furthermore, membrane-tethered Dock has a weaker phenotype than the untethered variant, arguing against the idea of membrane recruitment being an activation mechanism. As the manuscript focuses on Dock's interactions with WASp and Arp2/3 complexes, I found the section of *msn* out of place.
4. What is the significance of multinucleated egg chamber in Dock overexpression? Does this suggest a defect in cytokinesis when Dock is overexpressed?
5. The recruitment of WASp to the vicinity of ring canals in Dock overexpression is quite hard to see. In addition, what is the reason of using *MTD-GAL4* for Figure 5A, but *Mat Tub-GAL4*

© 2021. Published by The Company of Biologists under the terms of the Creative Commons Attribution License (https://creativecommons.org/licenses/by/4.0/).

for other Dock overexpression?

Minor comments:
1. The authors use heterozygous *dock* mutation in combination with *MTD>dock*^{RNAi} to deplete Dock protein in oocytes. Is this necessary? What are the phenotypes of *MTD>dock*^{RNAi} alone?
2. In Figure 3B, is the HA signal in control oocyte background?
3. Is Figure 3C mis-labeled with “GAL80^{ts}”?
4. I am confused with the ratio numbers in Figure 4B. The expression levels of *dock* mutants relative to tubulin seem higher than the HA-Dock lane.

Reviewer 2

Advance summary and potential significance to field

Cytokinesis, the process of cell cleavage, leads to the physical separation of the two daughter cells. However, in certain developmental contexts and specific tissues, cytokinesis is incomplete and the stalled cleavage furrow transforms into a stable intercellular bridge through the formation and maintenance of an actin-based structure called ring canal. These bridges facilitate intercellular communication, cell cycle synchrony and the transport of whole organelles. This process is well conserved in the male and female germline and has been observed in species ranging from insects to humans. Drosophila female oogenesis has been a prime model for decades to study the biogenesis of ring canals. In the Drosophila female germline, ring canals form at the end of each fourth cystoblast division and connect the 15 nurse cells to the oocyte. The nurse cells produce the necessary material that transit through ring canals for oocyte maturation. During the course of oogenesis, the ring canal diameter expands up to 20-fold in size. Numerous proteins are involved in its composition, including filamentous actin and Hts-RC (the product of the hu li tai shao gene). Moreover, its stable anchoring to the plasma membrane during nurse cell growth and oocyte maturation requires sustained E-Cadherin-dependent cell-cell adhesion. However, may aspects of ring canal biogenesis remain unknown, notably how ring canals form from the stalled cytokinesis ring and how their maturation is coordinated with cell growth.

This study from Stark et al., investigates the role and regulation of the adaptor protein Dreadlock (Dock) in the process of ring canal formation and maintenance in the drosophila female germline, and attempts to position Dock within the signaling pathway involved in ring canal formation. Dock, the ortholog of the well-studied vertebrate Nck adaptor protein, is known for its role in Drosophila male germline ring canal formation. In this study, Stark et al., show that Dock localizes to the female germline ring canals throughout oogenesis. They then assessed the consequences of attenuating or increasing WT or mutant Dock levels on ring canal integrity and oogenesis. They found that the modulation of Dock levels affects the integrity of ring canals and consequently oogenesis. In addition, they found subtle genetic interactions between Dock and the actin nucleation regulators WASp and the Arp2/3 complex, suggesting that Dock functions at ring canals by modulating F-actin dynamics.

Comments for the author

The authors have preformed a large body of work with careful quantification and made some interesting observations. However, I do not think that, at this stage, this work provides sufficient novel insights into the function and regulation of Dock on the biogenesis of ring canals to warrant publication in JCS.

Below are some specific points:

The introduction does not provide sufficient information on Dock/Nck function (although Nck has been extensively studied in a variety of cellular processes over the past 30 years), nor on what is known about the mechanism of ring canal biogenesis and maturation. This makes it difficult to assess the pertinence of the experiments and the novelty of the findings reported in this study. It also makes it challenging for non-specialists to understand. For example, in their experiments they use anti-HTS-RC antibodies to study the morphology of ring canals in various genetic backgrounds. However, no description of what HTS-RC is nor how it is involved in ring canal biogenesis and

© 2021. Published by The Company of Biologists under the terms of the Creative Commons Attribution License (https://creativecommons.org/licenses/by/4.0/).

maturation is provided in the introduction or in the result sections other than “it localizes in the inner rim of the ring”.

Different maternal promoters are used to drive the various RNAi and protein constructs through the UAS/Gal4 system. A major concern is that these promoters are activated at different stages of oogenesis, and, hence, at different stages of ring canal formation and maturation. Therefore it is difficult to assess which aspects of ring canal biogenesis are affected, making it difficult to compare the different results.

The rationale for most of the experiments are not clear and some conclusions are not supported by any representative figures. For example, in the results section the authors recurrently refer to “ring canal normal structure” (figure 1, figure 4C). However, a definition of “normal structure” is not provided. Another example is the conclusion that “Myr-Dock over-expression caused ring collapse and the formation of multinucleated cells.” However, the images presented in the figure (figure 3A and B) are too small to warrant this strong conclusion.

The authors associate the decrease in the number of ring canals per egg chamber (figure 4E) upon over-expression of a Dock SH3 domain mutant as a multinucleation phenotype, however, it is not possible to assess the multinucleation phenotype in egg chambers in the images provided in Figure S2B. Therefore, it is not currently possible to rule out that the decrease in ring canal numbers is due to cytokinesis proceeding to completion during some of the cystoblast divisions.

In summary, I do not feel that the manuscript warrants publication in JCS given these major concerns about the way in which experiments have been designed and interpreted and given the limited mechanistic insights that they provides.

Reviewer 3

Advance summary and potential significance to field

This well written manuscript by Stark, Crowe and Lewellyn describes a careful and novel analysis of the adaptor protein and mammalian Nck homolog, Dreadlocks (Dock), in the important model system of the Drosophila female germline. Dock has previously been studied in the context of the Drosophila male germline, while this is the first characterization of it's localization and role at ring canals during oogenesis. These ring canals originate from incomplete cytokinesis (arrested contractile rings), which retain F-actin and progressively reopen as oogenesis progresses. They serve as conduits for the transfer of cytoplasm from the nurse cells to the oocyte.

Immunofluoresence data are presented to clearly show endogenous Dock localization at ring canals throughout oogenesis, with a decline in immunostaining in stage 10 egg chambers. Reduction of Dock levels using RNAi (in a heterozygous mutant background) or germline mutant clones, is shown to have relatively mild, but clear, phenotypes of altered ring canal diameters (larger early and smaller later) and reduced levels of F-actin and another ring canal component, HtsRC.

Interesting perturbations of ring canal structures are documented upon over-expression of HA-Dock (or a weaker membrane-tethered HA-myr-Dock). A structure-function approach is then employed to probe mutations within each or all of Dock's 3 SH3 domains or its SH2 domain. This leads to the conclusions that the SH2 domain (and Src64) are required for Dock localization to ring canals (as previously shown in male RCs), while the SH3 domain(s) are required for the over-expression-induced defects in RCs. Genetic data are then presented to support the idea that WASp and Arp2/3 complex are involved in the Dock over-expression phenotypes, and a model is proposed in which Dock recruits WASp to activate Arp2/3 to control RC size.

Overall, the data are of high quality, well quantified, and support the claims made. I believe that this manuscript constitutes an interesting and important body of work that advances our mechanistic understanding of an important area of cell biology. It will be of interest to the readership of the Journal of Cell Science. I therefore recommend publication.

© 2021. Published by The Company of Biologists under the terms of the Creative Commons Attribution License (https://creativecommons.org/licenses/by/4.0/).

Comments for the author

Minor comments:
Why are Dock mutant RC diameters larger than controls in stage 5 then smaller in stage 6 onwards (Fig. 2)? Could this be more explicitly discussed? Could larger RCs at stage 5 represent incomplete closure or premature growth, and smaller ones later delayed growth?

Similarly, how does RC circumference correlate with the fluorescence intensities of F-actin and HtsRC (Fig. 2), i.e. how do the intensities per total RC length (as opposed to unit length) change? This is not strictly necessary to include, but it seems like it might offer additional mechanistic insight.

There is a typo at the bottom of Pg. 10: oogeneis

First revision

Author response to reviewers' comments

Response to Reviewer #1

Major issues:
1. Compared to the dock RNAi-knock downed oocytes, dock germline clones showed a stronger and more consistent reduction in ring canal size. The authors explain this discrepancy by demonstrating the presence of residual Dock proteins in the RNAied oocytes. However, another possibility is that the FRT, dock04723 chromosome contains a secondary mutation, which causes ring canal size reduction. Given the importance of dock loss-of-function phenotype, this should be addressed by additional experiments. The fact that dock overexpression has severe oogenesis phenotype may complicate a rescue experiment. How about using a different dock allele to rule out the possibility of background mutation in the dock04723?

We were able to acquire two additional dock mutant alleles – dockk13421 and dockW362. We were not able to perform the same analysis with the ovoD1 dominant female sterile line. Instead, we used a hs-FLP; ubi GFP FRT40 to make mosaic egg chambers. In this experiment, GFP marked the wild type and heterozygous mutant cells, and the homozygous mutant cells lack GFP. After multiple rounds of crosses, dissections, and staining, we obtained very few dockW362/dockW362 mutant nurse cells. We were more successful at obtaining mutant clones with the other allele, dockk13421, and we were able to generate the graph shown in Fig. S1B. We found that compared to egg chambers dissected from sibling controls who inherited the balancer chromosome (hs-FLP; ubi GFP FRT40/Cyo), ring canals connecting nurse cells that were GFP+ (and likely were heterozygous for the dockk13421 allele) were significantly smaller. Further, the ring canals connecting nurse cells lacking GFP (and were homozygous for the dockk13421 allele) were significantly smaller than ring canals in both the control and heterozygous mutant nurse cells. Therefore, we are confident that this reduced ring canal diameter observed in the mutant is due to the role of Dock in promoting ring canal growth. We added the following statement to the results section:

A similar decrease in ring canal diameter was also observed for ring canals connecting nurse cells homozygous for another mutation, dockk13421 (Fig. S1B).

2. Oocytes overexpressing dock exhibited severely disrupted ring canals, which is not the opposite of the loss-of-function phenotype (dock germline clones showed smaller ring canals, but unaffected ring canal morphology and composition). Even in the myr-dock overexpression, which has milder defects, it is not clear that the ring canal sizes are affected. The authors put forth an explanation that the dock protein level needs to be tightly regulated to control ring canal size. Given the importance of Dock protein level, is it possible to estimate the level of HA-Dock overexpression in MatαTub>HA-Dock relative to endogenous Dock?

© 2021. Published by The Company of Biologists under the terms of the Creative Commons Attribution License (https://creativecommons.org/licenses/by/4.0/).

We have performed a Western blot to determine the level of over-expression of Dock in the HA-Dock and Myr-HA-Dock experiments. We found that there was approximately a 13-20-fold increase in the levels of the longer isoform of Dock (indicated by the arrow) in ovary extracts. These data are included in Fig. S2A. We did also notice a prominent band in the Western blots that may correspond to a smaller Dock isoform – isoform A. We did most of our over-expression experiments using the longer form of Dock (corresponding to isoforms B, C, and D), but we did include data from an additional experiment where we over-expressed HA-Dock isoform A. This experiment showed that although over-expression of isoform A did produce some ring canal defects, the phenotype was much less severe than expression of the longer isoform. Further, this shorter isoform A did not strongly localize to the germline ring canals (Fig. S2B). We have added an additional paragraph to the discussion to address the potential role of the shorter Dock isoform.

3. The phenotypic similarities between Dock overexpression and myr-Msn suggest these two genes interact in ring canal formation. Consistent with this, the authors provide evidence that Msn and Dock recruit each other to nurse cell membrane.
However, the genetic interaction data between the two were negative.
Furthermore, membrane-tethered Dock has a weaker phenotype than the untethered variant, arguing against the idea of membrane recruitment being an activation mechanism. As the manuscript focuses on Dock's interactions with WASp and Arp2/3 complexes, I found the section of msn out of place.

Although we did not find a genetic interaction between Dock and Msn, because these two have been shown to physically and genetically interact in multiple other developmental contexts, and both localize to the ring canals, we felt that it was important to present this data in the paper. However, we have moved the localization data to Fig. S2C,D. We have also shortened the part of the discussion related to the Dock/Msn interaction to allow for more emphasis on the positive data we collected.

4. What is the significance of multinucleated egg chamber in Dock overexpression?
Does this suggest a defect in cytokinesis when Dock is overexpressed?

In the paper, we performed the Dock over-expression using two different GAL4 drivers. In Fig. 3, we used the MatαTub-GAL4 driver, which does not begin to show expression of GAL4 until approximately stage 2, which is after the mitotic divisions have completed (Hudson 2014). We went back to these images and counted the total number of HtsRC labeled ring canals in the earlier stages (stage 5 and younger). HtsRC is not recruited until after mitosis has been completed and therefore should mark the stabilized intercellular bridge structures. In the HA-Dock expressing egg chambers from the experiment in Fig. 3, we found that 71% of stage 3, 83% of stage 4, and 67% of stage 5 egg chambers imaged contained 15 visible ring canals. Many of the ring canals were detached from the nurse cell membrane or abnormally shaped. Therefore, although we cannot rule out a cytokinesis defect, we believe that in most cases, the germline cysts are formed with the correct number of nurse cells and ring canals, but those ring canals become unstable, failed to grow, or may even over-grow, which then leads to multinucleation. In the future, we hope to be able to visualize these phenotypes developing in live egg chambers to better understand the progression of the phenotypes observed. The following was added to the results section to address this issue:

We found that 67-83% of stage 3-5 egg chambers contained 15 ring canals labeled by HtsRC, which is recruited after the end of the mitotic divisions. Further because this GAL4 that is not expressed until after the mitotic divisions are complete, it suggests that the strong phenotype is not due to defects in incomplete cytokinesis, but that increasing the amount of Dock protein after ring canal formation has catastrophic consequences in the germline.

In Fig. 4, we did use the MTD-GAL4 driver, which shows expression of GAL4 beginning in the germarium and could therefore impact the mitotic divisions. In HA-Dock expressing egg chambers, 79% of stage 4 and 72% of stage 5 egg chambers imaged contained 15 visible ring canals. In HA-Dock SH3-1 expressing egg chambers, 64% of stage 4 and 86% of stage 5 egg chambers imaged contained 15 visible ring canals. In HA-Dock SH3-2 expressing egg chambers, 85% of stage 4 and 80% of stage 5 egg chambers imaged contained 15 visible ring canals. In HA-Dock SH3-3 expressing egg chambers,

© 2021. Published by The Company of Biologists under the terms of the Creative Commons Attribution License (https://creativecommons.org/licenses/by/4.0/).

90% of stage 4, and 89% of stage 5 egg chambers imaged contained 15 visible ring canals. We did find that using the MTD-GAL4, expression of HA-Dock did lead to a higher number of egg chambers that contained more than 15 nurse cells (and ring canals). Therefore, in the future, it would be interesting to further explore whether over-expression of HA-Dock in the germline could lead to packaging defects, where more than one cyst is encapsulated, or additional rounds of mitosis. The following sections have been added to the results/discussion to address this issue:

64-90% of stage 4-5 egg chambers expressing HA-Dock, HA-Dock SH3-1, HA-Dock SH3-2, or HA-Dock SH3-3 contained 15 ring canals marked by HtsRC. However, because we used MTD-GAL4, which expresses GAL4 throughout oogenesis, we cannot rule out that an earlier defect in cytokinesis could be contributing to these phenotypes.

It will also be of interest to further explore the progression of the Dock over-expression phenotype. When HA-Dock was expressed after the completion of germ cell divisions using Matαtub-GAL4 (Fig. 3), the observed phenotypes likely arose through destabilization of ring canals after they had formed, but the mechanism underlying this phenotype remains unclear. However, when HA-Dock was expressed using MTD-GAL4, there were a number of egg chambers that contained more than the expected 15 nurse cells; this phenotype was not observed in egg chambers expressing any of the other HA-Dock transgenes using MTD-GAL4. Futher studies will be required to determine whether this is due to a packaging defect or misregulation of germ cell divisions within the germarium.

5. The recruitment of WASp to the vicinity of ring canals in Dock overexpression is quite hard to see.

We have added arrowheads to indicate the modest recruitment of WASp near actin that has accumulated near abnormally shaped ring canals, but we agree with the reviewer that this enrichment is not as strong as we would have hoped to see. We have tried this staining under a number of different conditions, and we have also tried to visualize WASp using the MIMIC-WASp line, but we have never seen a stronger enrichment of WASp in any of these conditions. It is possible that this localization is weak or transient, which may make it more challenging to observe in fixed samples. Despite this, we find the genetic data to be compelling enough to suggest that this is at least part of the mechanism through which Dock acts in the germline.

In addition, what is the reason of using MTD-GAL4 for Figure 5A, but MatαTub-GAL4 for other Dock overexpression?

We used the MatαTub-GAL4 for the HA-Dock and Myr-HA-Dock lines that were initially generated using random p-element insertion (pPW Gateway plasmid), which was what we had available in the lab at that time. We did test these lines with the MTD-GAL4, but the phenotypes were so strong that they were hard to interpret, which is why we turned to the MatαTub-GAL4. Once we observed the strong phenotype with the HA-Dock line and decided to dissect the role of the various protein-protein interaction domains in this phenotype, we re-made the HA-Dock as well as each of the domain mutants in a plasmid (obtained from the Cooley lab) that would allow us to use the PhiC31 integrase to insert each of the transgenes in the same chromosomal location. The strength of the HA-Dock phenotype was weaker for the new PhiC31 line compared to the initial line made through p-element insertion, so we went back to the MTD-GAL4 line for experiments using these new transgenes. In addition, with the SH3-1,2,3 and SH2 mutants, we wanted to use the strongest driver possible to ensure that we were not over-looking a phenotype due to lower expression levels. We have added additional explanation in the materials and methods section to justify these choices, and we have further qualified the conclusions that can be drawn from each experiment.

The phenotypes observed in egg chambers expressing the HA-Dock transgene from the attP2 site (YS041 plasmid) were weaker than in egg chambers expressing the HA-Dock transgene integrated by random p-element insertion (pPW plasmid). Therefore, we used the stronger MTD-GAL4 (Fig. 4, 5A, S2B, S3, S4A) to express the various UAS-HA-Dock transgenes from the attP2 site throughout oognesis, and we used Matαtub-GAL4 to express the UAS-HA-Dock or UAS-Myr-HA-Dock beginning at stage 2 for the lines generated through p-element insertion (Fig. 3, S2A,D).

© 2021. Published by The Company of Biologists under the terms of the Creative Commons Attribution License (https://creativecommons.org/licenses/by/4.0/).

Minor comments:
1. The authors use heterozygous dock mutation in combination with MTD>dockRNAi to deplete Dock protein in oocytes. Is this necessary? What are the phenotypes of MTD>dockRNAi alone?

We have seen a similar modest increase in ring canal diameter in the dock-RNAi alone using either MTD-GAL4, MatαTub-GAL4, or nos-GAL4, but we were trying to increase the strength of the depletion phenotype to determine whether we would be able to see any more dramatic changes in ring canal size or stability. We added the following statement in the results section:

We have seen a similar increase in ring canal diameter using the UAS-dock-RNAi transgene alone with multiple different GAL4s (data not shown)…

2. In Figure 3B, is the HA signal in control oocyte background?

Yes, we assume that this HA signal in the oocyte is background, since we did not introduce any HA transgene into those control flies.

3. Is Figure 3C mis-labeled with "GAL80ts"?

We did use the Gal80ts in the background because that was the driver used to express these Myr-Msn transgenes in a previous publication (Kline 2018). This set of panels is now in Fig. S2C, and all panels in this figure now have clear labels to indicate the GAL4 used.

4. I am confused with the ratio numbers in Figure 4B. The expression levels of dock mutants relative to tubulin seem higher than the HA-Dock lane.

We went back and used the FluorChemQ (Protein Simple) software to quantify the intensity of the bands on the Western blot, and we have modified the relative expression of the various Dock proteins.

Response to Reviewer #2

Below are some specific points:

The introduction does not provide sufficient information on Dock/Nck function (although Nck has been extensively studied in a variety of cellular processes over the past 30 years), nor on what is known about the mechanism of ring canal biogenesis and maturation. This makes it difficult to assess the pertinence of the experiments and the novelty of the findings reported in this study. It also makes it challenging for non-specialists to understand. For example, in their experiments they use anti-HTS-RC antibodies to study the morphology of ring canals in various genetic backgrounds. However, no description of what HTS-RC is nor how it is involved in ring canal biogenesis and maturation is provided in the introduction or in the result sections other than "it localizes in the inner rim of the ring".

We have added to the introduction in order to highlight what is known about the role of Dock and Nck in other contexts as well as to provide a more complete introduction to the processes involved in ring canal formation and growth. This section now reads as follows:

About a dozen proteins have been identified that localize to the germline ring canals and regulate their size or stability (Yamashita, 2018). These proteins are organized into an inner and outer rim structure, which must be maintained and anchored to the nurse cell membrane while the ring canals grow. Actin is an abundant component of the inner rim, and during oogenesis, there is a dramatic increase in the number of actin filaments that is correlated with the significant growth in ring canal diameter (Tilney, 1996). The increase in actin filament number and their dynamic behavior likely requires the coordination of multiple actin nucleators and actin binding proteins, such as HtsRC, Kelch, and Cheerio (Gerdes et al., 2020; Hudson and Cooley, 2002; Kelso et al., 2002; Robinson et al., 1997; Robinson and Cooley, 1996; Thestrup et al., 2020; Zallen et al., 2002). In addition to actin and actin regulators, a number of kinases are known to localize to ring canals and/or regulate their growth (Dodson et al., 1998; Guarnieri et al., 1998; Hamada-Kawaguchi et

© 2021. Published by The Company of Biologists under the terms of the Creative Commons Attribution License (https://creativecommons.org/licenses/by/4.0/).

al., 2015; Kelso et al., 2002; Kline et al., 2018; Lu et al., 2004; O'Reilly et al., 2006). Two kinases, Btk29 and Misshapen (Msn), have been shown to impact the localization or modification of adherens junction proteins (Hamada-Kawaguchi et al., 2015; Kline et al., 2018), and a number of mutants that impact adherens junction turnover or the endocytic process lead to ring canal detachment and multinucleation (Bogard et al., 2007; Coutelis and Ephrussi, 2007; Langevin et al., 2005; Loyer et al., 2015; Murthy et al., 2005; Murthy and Schwarz, 2004; Oda et al., 1997; Peifer et al., 1993; Tan et al., 2014; Vaccari et al., 2009). This suggests that changes in the actin cytoskeleton, adherens junctions, and membrane trafficking are necessary to maintain ring canal anchoring while also facilitating growth, but how these processes are coordinated during oogenesis is not known.
An attractive candidate to integrate multiple pathways in the germline is the SH2/SH3 adaptor protein, Dreadlocks (Dock). Dock and its homolog, Nck, have been implicated in regulation of the actin cytoskeleton, endocytosis, membrane trafficking, and adhesion in many contexts, including immune cell activation and function, cell migration, cancer cell proliferation and invasion, axon guidance and targeting, and cell fusion (Abdallah et al., 2013; Buvall et al., 2013; Chaki and Rivera, 2013; Clemens et al., 1996; Ditlev et al., 2012; Garrity et al., 1996; Joseph et al., 2014; Kaipa et al., 2013; Martin et al., 2020; Ngoenkam et al., 2014; Rivera et al., 2004; Rohatgi et al., 2001). Further, Dock was shown to localize to germline ring canals during spermatogenesis in the fly (Abdallah et al., 2013). A number of Dock/Nck interacting proteins have been identified in other developmental contexts or in different cell types (Chaki et al., 2015; Fan et al., 2003; Hing et al., 1999; Kaipa et al., 2013; Li et al., 2001; Liu et al., 1999; Rivero-Lezcano et al., 1995; Rohatgi et al., 2001; Ruan et al., 1999; Takeuchi et al., 2010; Worby et al., 2001), but a role for Dock in the germline of the developing egg chamber has not been explored. If some of these interactions are conserved, it could place Dock in the unique position to coordinate changes in the actin cytoskeleton with changes in cell-cell adhesion or membrane trafficking to regulate ring canal growth while maintaining stability.

In the introduction and within the results and discussion, we have highlighted that HtsRC is an actin binding protein that is both necessary and sufficient to recruit actin to ring canals and regulate ring canal size. For example:

Egg chambers were stained with an antibody to HtsRC, which is both necessary and sufficient to recruit f-actin to the inner rim in order to regulate ring canal size and stability (Gerdes et al., 2020; Robinson et al., 1994; Yue and Spradling, 1992)

Different maternal promoters are used to drive the various RNAi and protein constructs through the UAS/Gal4 system. A major concern is that these promoters are activated at different stages of oogenesis, and, hence, at different stages of ring canal formation and maturation. Therefore it is difficult to assess which aspects of ring canal biogenesis are affected, making it difficult to compare the different results.

As mentioned in the response to Reviewer #1 above, we added additional description in the materials and methods section as well as at various points in the results and discussion to further address this point and emphasize when the transgenes were expressed and what can be concluded from each experiment. We have also added information on most figures to indicate which drivers were used in each experiment.

The rationale for most of the experiments are not clear and some conclusions are not supported by any representative figures. For example, in the results section the authors recurrently refer to "ring canal normal structure" (figure 1, figure 4C). However, a definition of "normal structure" is not provided.

We have included a description of normal ring canal structure:

To our surprise, depletion of Dock under these conditions did not cause any obvious defects in ring canal structure or integrity (Fig. 1D, 2A); the ring canals always contained a clear lumen and there were no obvious changes to the shape or structure compared to controls.

Additionally, when describing Fig. 4, we have added the description below:

© 2021. Published by The Company of Biologists under the terms of the Creative Commons Attribution License (https://creativecommons.org/licenses/by/4.0/).

The egg chambers always contained the expected number of ring canals (11 connecting nurse cells and 4 connecting the nurse cells to the oocyte), and those ring canals always contained a clear lumen that would presumably support cytoplasmic transfer to the oocyte.

Another example is the conclusion that “Myr-Dock over-expression caused ring collapse and the formation of multinucleated cells.” However, the images presented in the figure (figure 3A and B) are too small to warrant this strong conclusion.

We have added arrowheads to Fig. 3A to point to ring canals that are abnormally shaped or detached from the membrane, and we have provided an additional set of panels in Fig. 3B to better illustrate the phenotypes we observed.

The authors associate the decrease in the number of ring canals per egg chamber (figure 4E) upon over-expression of a Dock SH3 domain mutant as a multinucleation phenotype, however, it is not possible to assess the multinucleation phenotype in egg chambers in the images provided in Figure S2B. Therefore, it is not currently possible to rule out that the decrease in ring canal numbers is due to cytokinesis proceeding to completion during some of the cystoblast divisions.

See response to Reviewer #1 above.

Response to Reviewer #3

Minor comments:
Why are Dock mutant RC diameters larger than controls in stage 5 then smaller in stage 6 onwards (Fig. 2)? Could this be more explicitly discussed? Could larger RCs at stage 5 represent incomplete closure or premature growth, and smaller ones later delayed growth?

It is actually the dock04723/+; dock-RNAi condition that is larger than controls at stage 5, and the homozygous mutant condition is always smaller than controls and the RNAi condition. However, we have revised the discussion to more explicitly address possible explanations for the observed differences in ring canal size among the various dock manipulations.

Dock likely promotes ring canal growth indirectly through activation of the Arp2/3 complex. Depletion of the Arp2/3 subunit, ArpC2, leads to the formation of small, lumen-less ring canals (Thestrup et al., 2020). Although depletion or mutation of Dock on its own never resulted in lumen-less ring canals, we found a significant reduction in both actin and HtsRC (Fig. 2), suggesting that Dock may regulate the activity of an actin nucleator or other type of actin regulator. Further, reducing Dock levels significantly enhanced a weak arpC2-RNAi phenotype (Fig. 7), which suggests that the two could function in the same pathway. Although the larger ring canals observed at earlier stages in the dock04723/+; dock-RNAi egg chambers (Fig. 2) were initially puzzling, it was reminiscent of aspects of the arpC2-RNAi phenotype. When ArpC2 was depleted in the germline, we saw an increase in ring canal diameter in the germarium; at later stages, for ring canals that did contain a clear lumen, the average diameter was often larger than controls (Thestrup et al., 2020). This suggests that the Arp2/3 complex could play an early role in promoting contractile ring closure during incomplete cytokinesis and/or limit ring canal growth during later stages. If Dock functions upstream of the Arp2/3 complex at multiple stages of oogenesis, this could explain the variation in ring canal size that we observe. Alternatively, reducing Dock levels could lead to modest reductions in Arp2/3 activity, which slightly destabilizes the ring canal, leading to expansion, whereas stronger reductions could inhibit growth. Depletion of the Dock homolog, Nck, led to reduced myosin activity (Chaki et al., 2013), so it is also possible that the increase in ring canal size is due to reduced myosin-based contractility. Additional work will be required to distinguish between these possibilities.

Similarly, how does RC circumference correlate with the fluorescence intensities of F-actin and HtsRC (Fig. 2), i.e. how do the intensities per total RC length (as opposed to unit length) change? This is not strictly necessary to include, but it seems like it might offer additional mechanistic insight.

© 2021. Published by The Company of Biologists under the terms of the Creative Commons Attribution License (https://creativecommons.org/licenses/by/4.0/).

This is an excellent suggestion, and although we were not able to address this for the revision, we hope to explore this method of analysis in the future.

There is a typo at the bottom of Pg. 10: oogeneis

Thank you for noticing this typo; it has been corrected.

Second decision letter

MS ID#: JOCES/2020/254730

MS TITLE: Precise levels of the adaptor protein, Dreadlocks, maintain the size and stability of germline ring canals

AUTHORS: Kara Stark, Olivia Crowe, and Lindsay Lewellyn
ARTICLE TYPE: Research Article

I am happy to tell you that your manuscript has been accepted for publication in Journal of Cell Science, pending standard ethics checks.

Reviewer 1

Advance summary and potential significance to field

This revised manuscript by Stark et al. exams the role of dreadlock (dock), the Drosophila homolog of the SH2 and SH3 domains-containing Nck adaptor protein, in the ring canal formation during oogenesis. Dock associates with ring canal during oogenesis, and this localization is mediated by its SH2 domain and Src64 tyrosine kinase. Loss of dock function, by RNAi or independent loss-of-function alleles, reduces the ring canal size and decreases the presence of actin and HtsRC proteins at ring canals. Dock overexpression causes a catastrophic disruption of ring canal morphology, and this effect is abrogated if the SH2 or all three SH3 domains are mutated. More importantly, this Dock overexpression phenotype is suppressed by mutations in WASp and arp3, suggesting that Dock overexpression disrupts ring canal integrity by recruiting actin nucleation factors. Indeed, Dock overexpression increases the presence of WASp proteins in the vicinity of ring canals. The connection between Dock and actin nucleation is further strengthened by the synthetic phenotype caused by dock mutation and arpC2 RNAi. Based on this, the authors argue that Dock is recruited to the ring canals via Src64-dependent phosphorylation, and Dock controls ring canal morphology by regulating Arp2/3-mediated actin nucleation. As the ring canals are important for gamete development, the findings here should be of interests to researchers in cell biology and developmental biology.

Comments for the author

In the revision, the authors have included ring canal size analysis from an independent dock allele, which is important in resolving the phenotypic differences between RNAi and germline mutant clones.

The level of Dock overexpression was quite high (>10 fold for both HA-Dock and Myr-HA-Dock), but the genetic interaction between dock and arpC2 was done with loss-of-function allele and RNAi. I do appreciate that the authors addressed the comments by including the quantification experiment.

The questions regarding cytokinesis and GAL4 drivers have been addressed in revised text. The labeling of some of the figures has been clarified.

© 2021. Published by The Company of Biologists under the terms of the Creative Commons Attribution License (https://creativecommons.org/licenses/by/4.0/).

Overall, the comments raised by my initial review have been satisfactorily addressed, and I will support publishing this manuscript in JCS.

Reviewer 3

Advance summary and potential significance to field

This well written manuscript by Stark, Crowe and Lewellyn describes a careful and novel analysis of the adaptor protein and mammalian Nck homolog, Dreadlocks (Dock), in the important model system of the Drosophila female germline. Dock has previously been studied in the context of the Drosophila male germline, while this is the first characterization of it's localization and role at ring canals during oogenesis. These ring canals originate from incomplete cytokinesis (arrested contractile rings), which retain F-actin and progressively reopen as oogenesis progresses. They serve as conduits for the transfer of cytoplasm from the nurse cells to the oocyte.

Immunofluoresence data are presented to clearly show endogenous Dock localization at ring canals throughout oogenesis, with a decline in immunostaining in stage 10 egg chambers. Reduction of Dock levels using RNAi (in a heterozygous mutant background) or germline mutant clones, is shown to have relatively mild, but clear, phenotypes of altered ring canal diameters (larger early and smaller later) and reduced levels of F-actin and another ring canal component, HtsRC.

Interesting perturbations of ring canal structures are documented upon over-expression of HA-Dock (or a weaker membrane-tethered HA-myr-Dock). A structure-function approach is then employed to probe mutations within each or all of Dock's 3 SH3 domains or its SH2 domain. This leads to the conclusions that the SH2 domain (and Src64) are required for Dock localization to ring canals (as previously shown in male RCs), while the SH3 domain(s) are required for the over-expression-induced defects in RCs. Genetic data are then presented to support the idea that WASp and Arp2/3 complex are involved in the Dock over-expression phenotypes, and a model is proposed in which Dock recruits WASp to activate Arp2/3 to control RC size.

Overall, the data are of high quality, well quantified, and support the claims made. I believe that this manuscript constitutes an interesting and important body of work that advances our mechanistic understanding of an important area of cell biology. It will be of interest to the readership of the Journal of Cell Science. I therefore recommend publication.

Comments for the author

The authors have satisfactorily addressed my comments, which were minor, and have improved the manuscript. The text highlighting was appreciated. I recommend publication.

© 2021. Published by The Company of Biologists under the terms of the Creative Commons Attribution License (https://creativecommons.org/licenses/by/4.0/).
